# Gaming My Way to Recovery: A Systematic Scoping Review of Digital Game Interventions for Young People's Mental Health Treatment and Promotion

**DOI:** 10.3389/fdgth.2022.814248

**Published:** 2022-04-07

**Authors:** Manuela Ferrari, Judith Sabetti, Sarah V. McIlwaine, Sahar Fazeli, S. M. Hani Sadati, Jai L. Shah, Suzanne Archie, Katherine M. Boydell, Shalini Lal, Joanna Henderson, Mario Alvarez-Jimenez, Neil Andersson, Rune Kristian Lundedal Nielsen, Jennifer A. Reynolds, Srividya N. Iyer

**Affiliations:** ^1^Department of Psychiatry, McGill University, Montreal, QC, Canada; ^2^Douglas Mental Health University Institute, Montreal, QC, Canada; ^3^Department of Epidemiology, Biostatistics, and Occupational Health, McGill University, Montreal, QC, Canada; ^4^Department of Integrated Studies in Education, McGill University, Montreal, QC, Canada; ^5^Department of Psychiatry and Behavioural Neurosciences, McMaster University, Hamilton, ON, Canada; ^6^Black Dog Institute and School of Psychiatry, University of New South Wales, Sydney, NSW, Australia; ^7^School of Rehabilitation, Faculty of Medicine, Université de Montréal, Montreal, QC, Canada; ^8^Health Innovation and Evaluation Hub, Université de Montréal Hospital Research Centre, Montreal, QC, Canada; ^9^Centre for Addiction and Mental Health, Toronto, ON, Canada; ^10^Orygen, Parkville, VIC, Australia; ^11^Centre for Youth Mental Health, The University of Melbourne, Parkville, VIC, Australia; ^12^Department of Family Medicine, McGill University, Montreal, QC, Canada; ^13^Centro de Investigación de Enfermedades Tropicales (CIET), Universidad Autónoma de Guerrero, Acapulco, Mexico; ^14^Centre for Computer Games Research, IT University of Copenhagen, Copenhagen, Denmark; ^15^Canadian Centre on Substance Abuse and Addiction, Ottawa, ON, Canada

**Keywords:** digital games, digital mental health interventions, video games, e-interventions, youth, youth and young adults, scoping review, stepped care

## Abstract

Nearly all young people use the internet daily. Many youth with mental health concerns, especially since the Covid-19 pandemic, are using this route to seek help, whether through digital mental health treatment, illness prevention tools, or supports for mental wellbeing. Videogames also have wide appeal among young people, including those who receive mental health services. This review identifies the literature on videogame interventions for young people, ages 12-29, and maps the data on game use by those with mental health and substance use problems, focusing on evidence for the capacity of games to support treatment in youth mental health services; how stakeholders are involved in developing or evaluating games; and any potential harms and ethical remedies identified. A systematic scoping review methodology was used to identify and assess relevant studies. A search of multiple databases identified a total of 8,733 articles. They were screened, and 49 studies testing 32 digital games retained. An adapted stepped care model, including four levels, or steps, based on illness manifestation and severity, was used as a conceptual framework for organizing target populations, mental health conditions and corresponding digital games, and study results. The 49 selected studies included: 10 studies (20.4%) on mental health promotion/prevention or education for undiagnosed youth (Step 0: 7 games); 6 studies (12.2%) on at-risk groups or suspected mental problems (Step 1: 5 games); 24 studies (49.0%) on mild to moderate mental conditions (Steps 2-3: 16 games); and 9 studies (18.4%) focused on severe and complex mental conditions (Step 4: 7 games). Two interventions were played by youth at more than one level of illness severity: the **SPARX** game (Steps 1, 2-3, 4) and **Dojo** (Steps 2-3 and 4), bringing the total game count to 35 with these repetitions. Findings support the potential integration of digital games in youth services based on study outcomes, user satisfaction, relatively high program retention rates and the potential usefulness of most games for mental health treatment or promotion/prevention. Most studies included stakeholder feedback, and involvement ratings were very high for seven games. Potential harms were not addressed in this body of research. This review provides an important initial repository and evaluation of videogames for use in clinical settings concerned with youth mental health.

## Introduction

International research identifies adolescence and early adulthood as critical periods for first-time mental illness (1–5), with potentially devastating consequences when education and other developmental activities are disrupted. Serious mental illness is associated with further risks for co-occurring physical illnesses, as well as poverty and homelessness (6). Adolescents and young adults have also been especially hard hit by deteriorating mental health during the Covid-19 pandemic (7–10). Yet, despite the risks and burdens of mental illness, many young people delay or avoid seeking mental health treatment (11) for reasons that are complex. They include difficulties recognizing symptoms (12), negative perceptions of mental health services and professionals (13), and concerns about the stigma of mental illness (14–16). Fewer than 20% of young people with mental health problems use mental health services at all (17, 18). Rather, some 70-80% have turned to online sources for mental health information or help with emotional problems (19, 20), preferring the privacy, easy access and greater control over help-seeking afforded by virtual mental health care (21).

With nearly all young people using the internet (1, 22), research interest in digital mental health interventions has accelerated in response to their mental health needs and preferences, particularly since the pandemic (8, 23). Digital technologies including telehealth, internet, virtual reality, artificial intelligence, smartphones, and videogames (24–28) have been evaluated, showing comparable effectiveness among them (26). Digital interventions using cognitive-behavioral therapy (CBT) are especially effective in treating depression and anxiety (24, 26, 29, 30). Moreover, digital game interventions are frequently used by youth recovering from psychosis (31), who are receptive to the use of technology for receiving mental health interventions (32). Digital game interventions also show promise for improving user engagement in mental health care (23, 33). Serious games, defined as computerized games for educational purposes or for changing experience or behavior patterns (34), are used as therapeutic tools in treating anxiety, depression, autism, post-traumatic stress disorder (PTSD), attention-deficit/ hyperactivity disorder (ADHD) and alcohol use (33, 35–40). Game interventions promote improved memory, attention span, problem-solving, emotion management and socialization (41), better information retention (42), and learning (43), while supporting behavior change (44). Research also supports the benefits of integrating patient/staff and peer-to-peer interaction, and stakeholder collaboration into game designs (45).

By contrast, some studies have identified links between videogame use and depression, aggression, addiction, and negative moods (46–48). The risks of excessive internet use have also intensified during the pandemic due to worldwide school closures and prolonged confinement of youth at home (48). Gaming disorder, identified in the World Health Organization *International Classification of Diseases, 11th Revision*, is characterized by extreme investment of time (8-12 h/day) in gaming to the detriment of real-world relationships, daily routines, and life responsibilities (49). Prevalence rates for gaming disorder among youth have also been estimated at 10-15% in Asian countries and 1-10% in Western countries (50). Research presenting a proper assessment of gaming disorder is at an early stage. Some researchers have identified theoretical and methodological issues (51), while others still believe that solid evidence on gaming disorder as anything but a symptom of other disorders has yet to be produced (52, 53).

Youth mental health services are a rapidly developing field with a focus on prevention, early identification, treatment innovation and service development (54–56). Amid the crisis provoked by Covid-19 and its disproportionate psychosocial effects for young people (57), opportunities exist to deploy and scale up digital services to support mental health for this population (58). Yet, competing perspectives also exist around videogame technology as a tool for mental health treatment and education. However, little is known about how games have been used in clinical mental health services for youth, the experiences of young service users, families and service providers with videogame interventions, or strategies to assess possible harms (33, 59, 60) and pathways to gaming disorder (61). This “gaming my way to recovery” review aims to identify the existing literature on digital games for youth and map the evidence on the broad aims of these games and their use by young people with a full range of mental health and substance use problems.

## Materials and Methods

### Research Questions

The age range for this study includes “youth” (ages 12-19) and “young adults” (ages 20-29) as defined in Canadian public health (62). Five research questions (RQ) are addressed:

1) How have digital game interventions for mental health promotion and treatment been implemented in youth mental health services? What barriers and facilitators affected their implementation?
2) What were the outcomes of implementing digital game interventions and what evidence exists for the capacity of digital games to support mental health services in caring for youth?
3) What is known about youth, family, and service provider involvement in the development and implementation of digital interventions in youth mental health services?
4) What is known about youth, family, and service provider involvement in the evaluation of digital interventions in youth mental health services?
5) What are the potential harms and ethical practices related to use of digital interventions in youth mental health services?

### Study Design

Scoping review methodology was chosen as best suited to knowledge synthesis involving an array of evidence, publication types and research approaches. The five-stage methodological framework for scoping reviews by Arksey and O'Malley (63) was used in developing the study protocol (IRRID: PRR1-10.2196/13834) (64) and search strategy. An additional consultation stage (65) was added to the framework so that insights about the research could be gathered from partners (youth mental health services) and knowledge users (youth experiencing mental health problems). The information presented in this manuscript follows the PRISMA Extension for Scoping Reviews (PRISMA-ScR) Checklist (66) (see Appendix 1 in Supplementary Material).

### Stepped Care as a Conceptual Framework

The stepped care model, used increasingly to guide clinical practice and evaluate complex interventions in community-based mental health programs (67–69), was chosen as the conceptual framework for the study. Model 1, published in the “gaming my way to recovery” protocol (64), presents an adapted version of the traditional stepped care model for categorizing target populations and mental health conditions. This model organizes mental health, mental conditions and illness according to four levels of mental health condition severity (or steps), from at-risk groups or those with suspected mental health problems (Step 1), mild-moderate mental health conditions (Steps 2-3), combining mild and moderate levels of severity that are not always easy to disaggregate, to severe mental illness (Step 4). Step 0 was added to the original stepped care model, to include population-based interventions for mental health prevention/promotion and education for asymptomatic youth. The second column in the model was reserved for corresponding digital game interventions (focus and types) and the third column for evidence on contributions to knowledge by digital games at each step, in terms of processes, impact, effectiveness, sustainability, equity, engagement, and ethical practices.

### Stakeholder Engagement

Members of mental health services participated in the design of this scoping review project which led to the development of the published protocol (64). Project team members included service providers and researchers from Canada, Australia, and Denmark, who represented several international youth mental health networks (ACCESS Open Minds, Youth Wellness Hubs Ontario, Frayme, Orygen, and the Black Dog Institute). An advisory group was also established, meeting weekly during the project. Meetings were facilitated by a young peer researcher and self-identified gamer with lived experience of mental illness. The advisory group provided feedback on the data extraction form and helped to adapt the *Ladder of Children's Participation* (70) for the tool used to assess user engagement in studies under review. The advisory group was also involved in project dissemination activities (e.g., webinars).

### Search Strategy

The search strategy was constructed in collaboration with a health services librarian and reviewed by a second librarian following the Peer Review of Electronic Search Strategies checklist (71). The 23 strings in the search included terms related to mental health, mental disorders and a range of common/serious conditions as listed in section Study Identification and Selection (e.g., depressive disorders, anxiety disorders, schizophrenia, psychotic disorders, alcohol and substance-related disorders, mood disorders); game-related terms (e.g., video games, serious games, virtual reality, gamification); and a section of keywords related to platforms (e.g., online, internet, web, digital or computer, phone, apps, console, handheld). Initial searches were performed using multiple databases (e.g., Ovid MEDLINE, EMBASE, PsycINFO, the Cochrane Library) from inception to November 30, 2018; the search was later updated to April 2021. Only published, peer-reviewed studies were included. Duplicate citations were removed using EndNote citation software (Clarivate Analytics). The search strategy was published with the study protocol (64).

### Study Identification and Selection

Study identification criteria were as follows: (1) digital game interventions delivered on any technical platform, including personal computers, consoles (handheld or not), mobile devices and virtual reality; (2) interventions targeting mental health or substance use disorders or symptomatology, including depression, bipolar disorders, anxiety disorders, obsessive-compulsive disorder (OCD), schizophrenia and related psychotic disorders, eating disorders, PTSD, ADHD, specific phobia and interventions focused on mental health prevention, promotion or education; and (3) studies using any quantitative, qualitative or mixed methodologies. Review articles were not subject to data extraction and synthesis but were reserved for secondary reference searches.

### Screening Process

The two-phase screening process included title-abstract and full-text reviews. First, four trained screeners, working in teams of two, independently assessed studies for inclusion in the review following a preliminary selection using the Rayyan (Qatar Computing Research Institute) screening tool (72). The following exclusion criteria were applied: (1) dissertations or theses; (2) articles with missing abstracts; (3) conference presentations; (4) interventions targeting physical illnesses (e.g., cancer, dementia, chronic pain); (5) interventions delivered by telemedicine; (6) board games or commercial videogames used for entertainment only. Specifically, commercial video games designed for entertainment were excluded, except where they were used in clinical samples for therapeutic purposes. Second, the two raters read the full texts of articles that met the inclusion/exclusion criteria and made a final selection. Differences in ratings were resolved by consensus, or by the project lead in cases where consensus could not be reached.

### Data Extraction and Synthesis

The four raters performed the data extraction, which was validated by senior researchers (MF, JS). A data extraction table was developed based on study characteristics and variables related to the research objective and questions. Data were extracted from articles in the final selection for the following categories: (1) study characteristics: aims/hypotheses, country, setting, methodology, theoretical framework/model, interventions, model and stakeholder participation ratings; (2) participant characteristics: age, sex, ethnicity; (3) game characteristics: object/description, type of game, digital platform, guidance (y/n), target diagnoses; (4) stakeholder engagement: ratings on participation in game development and evaluation by youth, family and service providers; (5) outcome measures; and (6) findings: quantitative, qualitative, player satisfaction, barriers/facilitators to implementation, adherence/attrition, and loss to follow-up (see Study Data file, Appendix 2 in Supplementary Material). Data were extracted for each article, synthesized, and entered by category for use in revising Model 1 (see the “gaming my way to recovery” protocol) (64).

## Results

### Search Results and Study Characteristics

**Figure 1:**
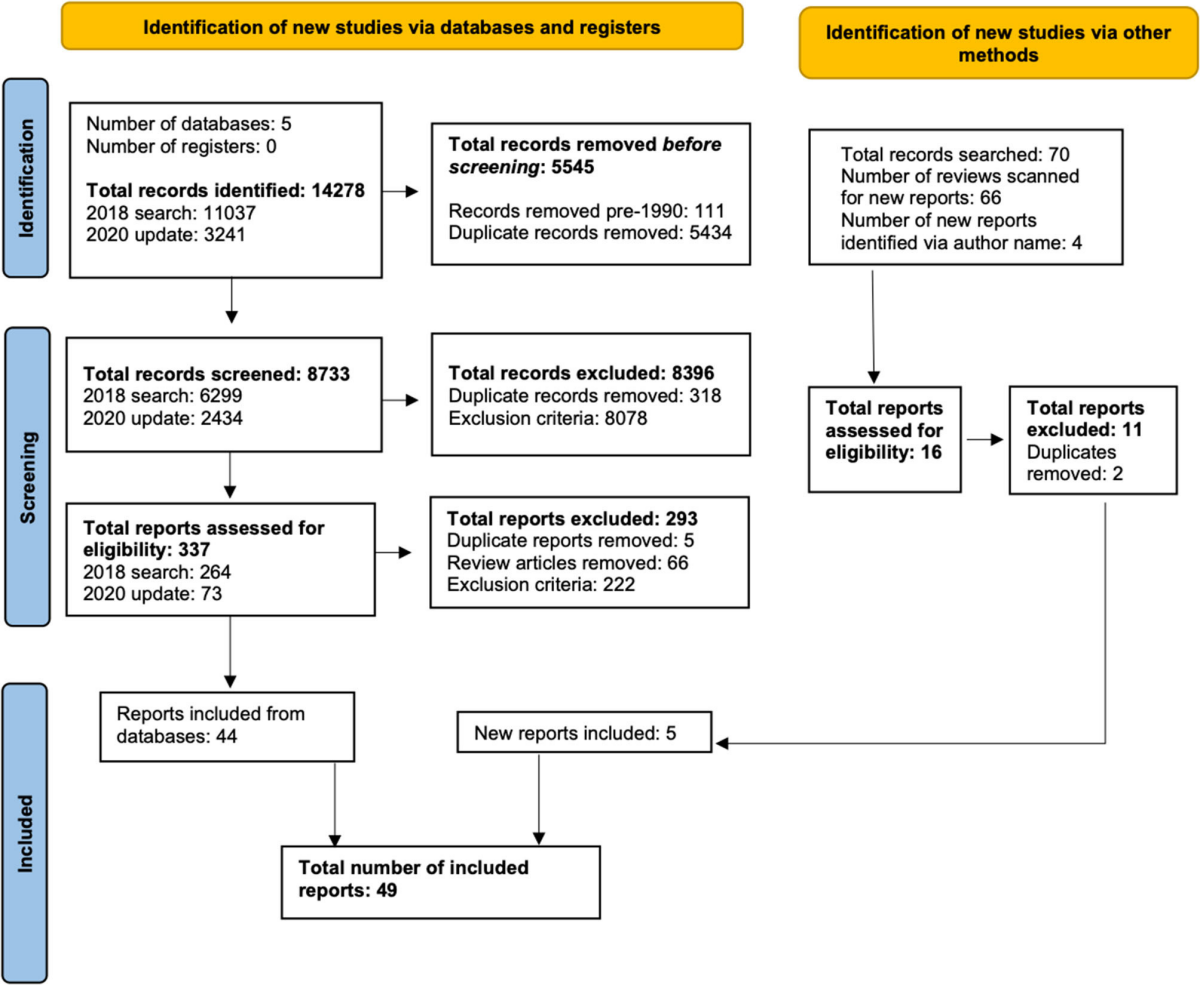
PRISMA 2020 flow diagram for updated systematic reviews which included searches of databases, registers, and other sources.

A PRISMA flow diagram (73) summarizing the search results was produced (see Figure 1). Of a total 8,733 articles screened from the original and updated searches, 40 met the inclusion criteria, four were identified in the updated search, and five were identified through other methods for a total of 49 studies in the final review. These studies represented 12 different countries: over half from the USA (*n* = 10), the Netherlands (*n* = 9) and New Zealand (*n* = 8); followed by Spain (*n* = 5), Ireland and Romania (four studies each), Australia, Hong Kong, and South Korea (two studies each) and Chile, India, and Germany (one study each) (see Study Data file, Appendix 2 in Supplementary Material).

### Participant Characteristics and Mental Health Conditions

The 49 studies included 6,592 participants in total. Of the 40 studies with more than one participant and that provided data on gender, 57% (*n* = 2,199) of participants were female and 43% (*n* = 1,680) male. Most studies (*n* = 30) focused on “youth” 19 years or younger. The mean age was 14.4 years for youth and 22.4 years for young adults. Of the 49 studies, 39 tested games for 15 different diagnoses, while 10 studies focused on mental wellness for undiagnosed youth. Depression/depressive symptoms ranked first as the focus of 13 studies (26.5%) followed by studies for depression/anxiety or multiple forms of anxiety (six studies: 12.2%); substance use disorder, and alcohol use disorder or hazardous drinking accounted for three studies each, while complex trauma and obsessive-compulsive disorder had two studies each. The remaining diagnoses, with one study each, were ADHD, bulimia nervosa, specific phobia, PTSD, first episode psychosis, self-identified, and mental health problems (see Study Data file, Appendix 2 in Supplementary Material).

### A New Stepped Care Model and Study Results

**Figure 2:**
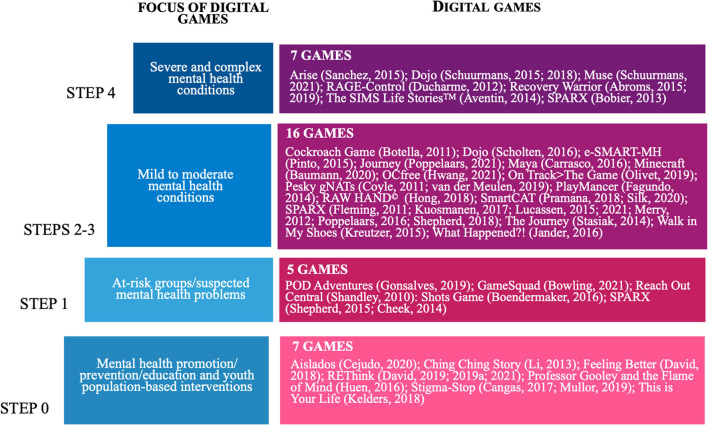
Stepped Care Model for Videogame Interventions.

The Stepped Care Model for Videogame Interventions (see Figure 2), developed for this study, distributes the 32 games identified in the review across four steps: Step 0 describing population studies for mental health prevention, promotion, and education with undiagnosed youth, while Steps 1, 2-3, and 4 described ascending severity levels for mental illness, from at-risk or suspected mental health problems, to mild-moderate mental health conditions, to severe and complex mental health conditions. **SPARX**, the game most studied (74–83), was played by youth at all three levels of illness severity (Steps 1, 2-3, 4) and **Dojo** (84–86) was played at Steps 2-3 and 4, which brings the total game count to 35 taking these repetitions into account. Other games tested in more than one study were: **Recovery Warrior** (87, 88) (Step 4), **SmartCAT** (89, 90) (Steps 2-3), **Pesky gNATs** (91, 92) (Steps 2-3), and **REThink** (93–96) (Step 0). The remaining games were evaluated in single studies. Table 1 provides a general overview of characteristics for each digital game intervention (e.g., language, genre, duration, game customization, storyline, and goals).

**Table 1:** Game characteristics.

**Game name**	**Language**	**Genre**	**Time limits**	**Game storyline**	**Character customization (Y/N)**	**Biofeedback (Y/N)**	**Motion–activated video game (Y/N)**	**Game website**
**Aislados**	Spanish	Role-playing game	55 min./session	Students visit a shipwreck, whose occupants end up lost on an island. Players control one of the characters and make decisions on how to deal with different situations.	Unclear	No	No	Not available
**Arise**	English	Puzzle game	10 min.	No storyline	No	No	No	Not available
**Ching Ching Story**	Chinese	Role-playing game	NA	Overall game has ten missions to be fulfilled [storyline not clear].	Unclear	No	No	Not available
**Cockroach Game**	Spanish	Puzzle game	15–35 min.	Two scenarios with different levels of difficulty. 1) user sees cockroaches on various surfaces displayed on the phone screen; the virtual insects wear (closed toe) shoes at the first level, summer shoes (open toe) at the intermediate level, and on a hand at the advanced level. 2) users see (on camera) virtual cockroaches on real surfaces (e.g., on their real clothes, hands, etc.).	No	No	No	Not available
**Dojo**	English	Adventure and puzzle game	30 min./session	Player discovers a hidden temple and is accompanied by Dojo masters through various trainings and challenges. The player must remain calm under stress.	No	Yes	Yes	YouTube demo: https://www.youtube.com/watch?v=WNV4A8R8v4A
**e-SMART-MH**	English	Role-playing game	15–20 min.	Young adult interacts with three-dimensional avatars of healthcare providers and staff in a virtual office visit. A virtual coach supports the young adult in communications with the healthcare team; gives tailored feedback.	Unclear	No	No	Not available
**Feeling Better**	Romanian and English	Action and adventure game	NA	Player selects a character and undertakes a flying challenge to collect functional emotions written on colorful balloons, while avoiding dysfunctional emotions and other obstacles.	Unclear	No	No	https://rethinkplatform.ro/
**GameSquad**	English	Exergames	NA	In Just Dance, players imitate on-screen dance choreography from over 40 songs, and are judged on their ability to follow a dance routine to a chosen song. Players can unlock gifts such as new songs, game modes, and Dance Mashups combining different dance routines.	No	No	No	YouTube link to Just Dance: https://www.youtube.com/watch?v=1S_Ch_qzWp8
**Journey**	None	Adventure game	Not clear	Players travel through a desert and underground areas to the top of a snowy mountain.	No	No	No	https://thatgamecompany.com/journey/#
**Maya**	Spanish	RPG adventure game	11:57 min.	Maya, a sad teenager, gets involved in an environmental protest with her friend to save a park. They give information to an official. The protesters win and save the park.	Unclear	No	No	Not available
**Minecraft**	None	Exploration game	10–20 min.	Two comparable, virtual environments: a pirate island (“island”), and a large mansion and surroundings for players to explore.	No	No	No	Not available
**Muse**	English (Dutch translation of tutorials)	Gamified smartphone app	5–20 min./session	A game-based meditation app with 10 relaxation tutorials that are played on an iPad. Players wear an electroencephalography (EEG)–based headband that provides real-time neurofeedback.	No	Yes	No	https://choosemuse.com/
**OCfree**	Korean	Quests, serious and educational games	NA	No storyline.	NA	No	No	Not available
**OnTrack>The Game**	English	Role-playing game	45-60 min.	Initial scene as players have moved in and need to unpack. Energy-consuming activities (e.g., unpacking, visiting new environments) delete an energy bar, which is restored by doing restorative activities (e.g., taking a shower, sleeping). Game teaches about causes, symptoms, and treatment of FEP through a computer. Characters visit other places where they can watch videos inspiring hope and recovery.	Yes	No	No	YouTube video: https://www.youtube.com/watch?v=c9WiJYCePNI
**Pesky gNATs**	English	Role-playing game	45-50 min.	A series of game characters introduce mental health concepts through conversation, embedded animations, videos, and questions about player's own situation. Players carry an in-game notebook for answering characters' questions and recording new ideas. Negative automatic thoughts are presented as little creatures called gNATs who sting people, causing 9 types of negative thinking. gNATs provide a concrete representation of a key CBT concept.	No	No	No	https://peskygnats.com/
**PlayMancer**	Spanish	Role-playing game	20 min.	Video game includes three mini-games: (1) The Face of Cronos, based on biofeedback; (2) Treasures of the Sea, a virtual swimming game that trains for visuospatial abilities, visual working memory, and decision making; and (3) Sign of the Magupta, a relaxation game.	Unclear	Yes	Yes	Not available
**POD Adventures**	English, Hindi, and Konkani	Adventure game	Not clear	Game teaches problem-solving skills through interactive animated vignettes and personalized action plans, with encouraging prompts and feedback by an in-app guide character. Storyline is not clear.	Unclear	No	No	https://appsonwindows.com/apk/10045950/
**Professor Gooley and the Flame of Mind**	Chinese	Role-playing and adventure game	45 min.	User takes on role of a space intern in a fictional setting showing cognitive distortions on earth. Guided by Professor Gooley, they undertake a space journey to 8 planets, searching for the Flame of Mind to solve the world health crisis. Quests prompt them to learn and apply psychological constructs to obtain the various components for activating the Flame of Mind.	Unclear	No	No	www.gooley.edu.hk
**RAGE-Control**	English	Action game (Space Invader style)	30–45 min. learning phase; 15 min. self-regulation phase	Like the arcade game, Space Invaders, with a pulse oximeter to measure heart rate. Player maneuvers a spaceship horizontally, firing at enemy spaceships, withholding fire when friendly spaceships fly past.	No	Yes	No	YouTube video: https://www.youtube.com/watch?v=X1s8D8FpG90
**RAW HAND**	English and Korean	Platform game	30 min./session	Players select one of five games in the platform and perform related tasks based on the concept of repetitive exposure and response prevention.	No	Yes	Yes	Not available
**Reach Out Central**	English	Role-playing game	69-91 min.	Player completes a short survey that measures positive affect, then is presented with game scenario around move to a new town. In-game mood (tracked over time) is affected by activities and response to characters and situations.	Unclear	No	No	Not available
**Recovery Warrior**	English	Body motion-activated game	NA	The suite of games makes use of whole-body motion detection and a voice-recognition feature. Drug refusal elicits the phrase “I'm clean”. Games include Recovery Ninja (destroy drugs), Recovery Ninja+Goodies (destroy and discern), Recovery Climber (avoid drugs), Recovery Racer (destroy drugs), Recovery Racer+Goodies (destroy and discern), Recovery Runner (avoid drugs), and Recovery Runner+Goodies (avoid and discern).	No	No	Yes	Not available
**REThink**	Romanian and English	Several genres, mainly action and adventure game	50 min.	Developed around the character of RETMAN, player practices Rational Emotive and Behavioral Therapy. RETMAN guides youth in saving the minds of Earth's inhabitants from the powers of Irrationalizer.	Unclear	No	No	https://rethinkplatform.ro/
**Shots Game**	Dutch	Gamified digital platform	Not clear	Game is like a slot machine; uses a coin-based reward system and attractive graphics, animations (e.g., spinning wheels with pictures of beverages), and sound effects.	No	No	No	Not available
**SmartCAT**	English	Platform game	2-5-min./ mini- game	Each game (of 4) contains content for multiple scenarios and levels of difficulty.	No	No	No	Not available
**SPARX** (SPARX-R and Rainbow SPARX)	English	Fantasy game	30 min./ module	In SPARX (Smart, Positive, Active, Realistic, X-factor thoughts), the young person chooses an avatar and undertakes a series of challenges to restore the balance in a fantasy world dominated by GNATs (Gloomy Negative Automatic Thoughts).	Yes	No	No	https://www.sparx.org.nz/home
**Stigma-Stop**	English and Spanish	Role-playing game	60 min.	Various scenarios.	No	No	Unclear	YouTube video: https://www.youtube.com/watch?v=tbr827Y4nvw
**The Journey**	English	Fantasy game	20-30 min./ module	Setting is a fantasy game environment, where user selects and names an avatar, undertakes a quest through magical lands. Themes are linked to content (e.g., cognitive restructuring techniques covered in Sky and Star Cities). Points earned for completing each of seven modules; rewards after each module.	No	No	No	Available on request from the author
**The SIMS Life Stories**™	English	Role-playing game	Not clear	Game follows the lives of Riley, who moves from SimCity to Four Corners to start a new life with her Aunt Sharon, and Vincent, a millionaire searching for true love. The towns in which Riley and Vincent live are also available for gameplay after completion of the stories.	Yes	No	No	https://sims.fandom.com/wiki/The_Sims_Life_Stories
**This is Your Life**	Dutch	Fantasy game	30 min.	User follows a journey toward a flourishing life, guided by a professor.	Unclear	No	No	Not available
**Walk in My Shoes**	English	Platform or narrative game	2–3 hours, entire game	No storyline	Unclear	No	No	Not available
**What Happened?!**	Dutch	Role-playing game	NA	In game sessions, the adolescent wakes up after a night of partying and does not remember what happened the night before. The 2-dimensional game leads the player to find out what happened.	Unclear	No	No	Not available

The new Model provides a comprehensive overview of the state of knowledge in youth digital game technologies, showing how games have been implemented, their outcomes and evidence of capacity for potential use in youth mental health services, based on seven key constructs:

- **Processes:** study methodologies, including implementation barriers and facilitators (research question (RQ) 1).
- **Impact:** identification of games with significant improvement on main variable(s) of interest (e.g., symptom reduction for specific or multiple mental health conditions, improved protective factors), and positive results on secondary variables (e.g., life satisfaction, quality of life, psychological functioning, stigma, etc.) (RQ2).
- **Effectiveness**: statistical ratings/magnitudes of effect or descriptions of effectiveness based on specific outcomes that met study objectives, or study conditions, game designs, delivery platforms, etc. described as “effective”; user satisfaction and acceptability ratings/descriptions (RQ2).
- **Sustainability:** maintenance of gains from baseline to post-intervention/follow-up periods; intervention scalability (RQ2).
- **Equity:** reach of game to underserved populations (e.g., sexual, cultural/ethnic minorities; youth from socially and economically disadvantaged and/or remote areas) (RQ2).
- **Engagement:** types of stakeholder participation (youth, families, service providers, outside experts) in game development, evaluation, and levels of involvement (RQ3, RQ4).
- **Ethical Practices**: specific measures taken to insure player privacy, confidentiality, and safety; harm reduction measures including attention to potential risks for gaming disorder (RQ5).

The data on these constructs are presented in Supplementary Table 1: Data for 49 studies using the Stepped Care Model for Videogame Interventions as a conceptual framework (see Appendix 3 in Supplementary Material). The findings under Engagement were described in study narratives but were also included in the results on user satisfaction and acceptability. Thus, the complete data on stakeholder participation in developing, evaluating, and testing the videogame interventions, and involvement scores for each game, are presented separately in Table 2 as well as definitions for the four levels of participation and scoring procedures. The complete study results are described below, from no mental health diagnosis (Step 0) to the three levels of mental illness severity (Steps 1-4).

**Table 2:** Participation data and scores on stakeholder involvement in developing, evaluating, and testing 32 videogame interventions^*^.

**Game name/** **Author**	**4 = partnership, collaboration (Game development; involvement in game delivery)**	**3 = consultation, feedback (Game evaluation)**	**2 = inform (game testing or delivery; study data collection on game experience or acceptability)**	**1 = gameplay by youth study participants; no participation**	Total scores**
**POD Adventures** Gonsalves et al. (97)	Co-design workshops with youth and service providers (4)	Youth consultation at each stage (3)	Service provider focus groups; youth game testing sessions (2)	Gameplay (1)	(10)
**SPARX** Shepherd et al. (79)	Youth, providers, and various Maori experts contributed to game design and development; youth included in co-design workshops (4 x 2)		Youth and family assessment and feedback (focus groups) (2)	None	(10)
**SmartCAT** Pramana et al. (89)	Therapists and youth involved in user-centered design (UCD approach) (4)		Youth satisfaction rated (2)	Gameplay (1)	(7)
**SPARX** Merry et al. (77)	Youth participation in game development (4)		Youth satisfaction rated (2)	Gameplay (1)	(7)
**SPARX** Lucassen et al. (81)	Collaboration between clinicians and youth in game development (4)		Youth satisfaction rated (2)	Gameplay (1)	(7)
**What Happened?!** Jander et al. (98)	Youth participation in game development (4)	Parent involvement in game assessment		Gameplay (1)	(5)
**SPARX** Cheek et al. (75)		Youth and family assessment and feedback (3)		Gameplay (1)	(4)
**Arise** Sanchez and Bartel (99)	5-member consultation board of addiction experts evaluated and revised an early prototype of game		Youth and treatment provider satisfaction rated (2)	Gameplay (1)	(3)
**SPARX** Kuosmanen et al. (76)		Parent involvement in game assessment	Youth game assessment (2)	Gameplay (1)	(3)
**SPARX** Fleming et al. (83)		Parent involvement in study recruitment	Youth satisfaction rated (2)	Gameplay (1)	(3)
**Stigma-Stop** Cangas et al. (100)			Youth survey on program (2)	Gameplay (1)	(3)
**Pesky gNATs** Coyle et al. (91)			Therapist in-study assessment; youth game assessment (2)	Gameplay (1)	(3)
**Maya** Carrasco (101)	Collaboration, contribution, and approval of game by outside experts (2 therapists and 2 psychotherapists); input from team at Heidelberg Center for Psychotherapy Research		Youth post-game assessment; therapist interviews on game as treatment tool (2)	Gameplay (1)	(3)
**REThink** David et al. (93)			Youth satisfaction rated (2)	Gameplay (1)	(3)
**RAGE-Control** Ducharme et al. (102)			Youth satisfaction rated (2)	Gameplay (1)	(3)
**This is Your Life** Kelders et al. (103)			Youth satisfaction rated (2)	Gameplay (1)	(3)
**Ching Ching Story** Li et al. (104)			Youth satisfaction rated (2)	Gameplay (1)	(3)
**OnTrack>The Game** Olivet et al. (105)			Youth and provider evaluations of game (2)	Gameplay (1)	(3)
**e-SMART-MH** Pinto et al. (106)			Youth rating of game elements, satisfaction, and acceptability (2)	Gameplay (1)	(3)
**SPARX** Poppelaars et al. (78)			Youth satisfaction rated (2)	Gameplay (1)	(3)
**SmartCAT** Silk et al. (90)			Youth and parent satisfaction rated (2)	Gameplay (1)	(3)
**Dojo** Schuurmans et al. (85)			Youth satisfaction and enjoyment rated (2)	Gameplay (1)	(3)
**Dojo** Schuurmans et al. (86)			Youth satisfaction rated (2)	Gameplay (1)	(3)
**Reach Out Central** Shandley et al. (107)			Youth satisfaction and enjoyment rated (2)	Gameplay (1)	(3)
**SPARX** Shepherd et al. (80)			Youth evaluation (esp. cultural elements) (2)	Gameplay (1)	(3)
**The Journey** Stasiak et al. (108)			Youth ratings on feasibility and acceptance (2)	Gameplay (1)	(3)
**Journey** Poppelaars et al. (109)			Youth game assessment (2)	Gameplay (1)	(3)
**OCfree** Hwang et al. (110)			Youth satisfaction survey (2)	Gameplay (1)	(3)
**REThink** David et al. (95)			Youth satisfaction assessed (2)	Gameplay (1)	(3)
**GameSquad** Bowling et al. (111)	Parents involved in advisory team that adapted GameSquad for youth with NPD	Parents involved in screening for study	Youth exit questionnaire with game assessment (2)	Gameplay; parent involvement in coaching sessions (1)	(3)
**Muse** Schuurmans et al. (112)			Youth game enjoyment assessed (2)	Gameplay (1)	(3)
**SPARX** Bobier et al. (74)			Youth game satisfaction assessed (2)	Gameplay (1)	(3)
**The SIMS Life Stories**™ Aventin et al. (113)		Residential social workers delivered game	Semi-structured interviews with social workers	Gameplay (1)	(1)
**Dojo** Scholten et al. (84)		Service providers involved in study recruitment		Gameplay (1)	(1)
**Pesky gNATs** van der Meulen et al. (92)			Therapist in-study evaluation of game	Gameplay (1)	(1)
**SPARX** Lucassen et al. (82)				Gameplay (multiple studies) (1)	(1)
**Cockroach Game** Botella et al. (114)				Gameplay (1)	(1)
**Aislados** Cejudo et al. (115)				Gameplay (1)	(1)
**Feeling Better** David et al. (96)				Gameplay (1)	(1)
**REThink** David et al. (94)				Gameplay (1)	(1)
**PlayMancer** Fagundo et al. (116)				Gameplay (1)	(1)
**RAW HAND** Hong et al. (117)				Gameplay (1)	(1)
**Professor Gooley and the Flame of Mind** Huen et al. (118)				Gameplay (1)	(1)
**Walk in My Shoes** Kreutzer and Bowers (119)				Gameplay (1)	(1)
**Minecraft** Baumann et al. (120)				Gameplay (1)	(1)
**Recovery Warrior** Abroms et al. (88)				Gameplay (1)	(1)
**Recovery Warrior** Abroms et al. (87)				Gameplay (1)	(1)
**Shots Game** Boendermaker et al. (121)				Gameplay (1)	(1)
**Stigma-Stop** Mullor et al. (122)				Gameplay (1)	(1)

*Levels of participation:

***4** **=** **Partnership/collaboration:** Involvement in game development activities, e.g., input into game development; involvement in co-design workshops; Advisory board membership; responsibility for revision/approval of game prototypes*.

***3** **=** **Consultation/feedback:** stakeholder involvement in game evaluation outside of study participation; unspecified consultation or feedback reported in narrative form; also, delivery of game by service providers and feedback; involvement in study recruitment or screening of potential participants*.

***2** **=** **Inform/game testing or delivery:** gameplay to test the intervention, includes in-game questionnaires or scales completed by youth, parents, service providers/therapists to rate game experience, acceptability, satisfaction, or feasibility*.

***1** **=** **Game play only, by youth, others:** No additional questionnaires or rating of the game experience*.

**Scoring procedure: Scores are based on the sum of results for levels 4 + 3 + 2 + 1. Only participation by youth, as end-users of the intervention, is scored. Participation by other stakeholder groups is described.

*In ranking games that were tested in two or more studies, the study with the highest score was selected as the best representation of youth participation for a given game intervention*.

#### Step 0: Mental Health Promotion/Prevention/Education and Youth Population-Based Interventions

The 10 studies (20.4% of the total 49) at Step 0 targeted young people without mental health diagnoses and included seven games focused on wellness through mental health promotion, prevention, and education. The five youth games involved mental health promotion and prevention: **Aislados** (115), **REThink** (93–95), **Professor Gooley and the Flame of Mind** (118), **Stigma-Stop** (100) for mental health education, and **Feeling Better** (96) on emotion regulation. The two games for young adults included a mental health prevention game, **This is Your Life** (103), and **Ching Ching Story** (104) for mental health education. **Sigma-Stop** (122), a third, was the same game as played by youth at Step 0.

##### Youth Games

Processes (RQ1) included two randomized clinical trials (93, 94), three pre-post game assessments (95, 100, 115), a pilot sequential game assessment (96) and a structural equation model (118). Barriers related to game design involved insufficient game duration and intensity in **Feeling Better** (96), viewed as a possible reason for results that did not meet expectations, and high attrition rates in **Professor Gooley and the Flame of Mind** (118) attributed to the self-help format. One study design/research issue in a pilot study concerned small sample size and lack of a control group (96).

Outcomes (RQ2) related to Impact showed significant improvement on the main variables of interest for all five games: for depressive mood in **Feeling Better** (96), health-related quality of life and mental health in **Aislados** (115), reduced stigma around mental illness in **Stigma-Stop** (100), improved functional emotion in **REThink** (93, 94) and user learning/psychological wellbeing in **Professor Gooley and the Flame of Mind** (118). Effectiveness was further demonstrated for **REThink** (93, 95), with a moderate effect on emotional symptoms but large effect on depressive mood. Regarding secondary measures, **Aislados** (115) promoted positive affect and better mental health. On user satisfaction/acceptability, **Stigma-Stop** (100) scored high on game usefulness, with 75% of players also recommending the game. Satisfaction scores at mid-intervention favored **REThink** (93) over controls. Study retention was reported for three games: **Feeling Better**, 88.0% (96); **REThink**, 86% (93, 94); and **Professor Gooley and the Flame of Mind**, 38.6% (118). However, on Sustainability, results were not sustained for **REThink** after three trials and game satisfaction faded by post-test (93, 96). Equity was promoted especially in **Professor Gooley and the Flame of Mind** (118), as all secondary students in Hong Kong were invited to the study.

Regarding Engagement (see Table 2), two youth games, **REThink** (93, 95) and **Stigma-Stop** (100) reported user satisfaction ratings, while these and the remaining youth studies, **Aislados** (115), **Professor Gooley and the Flame of Mind** (118), **Feeling Better** (96) and the other **REThink** study (94) included gameplay but no participation. The involvement ratings for these studies were: **Stigma-Stop** (100) and **REThink** (93, 95): 3 and for the games with no assessment: **Aislados**, **Professor Gooley and the Flame of Mind**, **Feeling Better**: 1 and a score of 1 for the remaining **REThink** study (94). Concerning Ethical Practices (RQ5), no results were reported.

##### Young Adult Games

Processes (RQ1) for these three studies included one experimental design (103) and two pre-post game assessments (104, 122). The one reported barrier involved a study design/research issue where lack of clarity in definitions of key concepts adversely affected the measures chosen for the study (103).

Outcomes (RQ2) on Impact showed significant improvement on the main variables of interest for all three studies: on game involvement and flow in the gamified vs. non-gamified condition in **This is Your Life** (103), mental health literacy in **Ching Ching Story** (104) and on all measures in **Stigma-Stop** (122): dangerousness, avoidance, segregation and anger. Effectiveness for the gamified version of **This is Your Life** (103) was equivalent to the non-gamified version on cognitive and affective engagement; while the **Ching Ching Story** (104) was most effective in enhancing mental health literacy. **Stigma-Stop** (122) demonstrated similar effectiveness as face-to-face contact or a talk by a professional in reducing stigma. Regarding user satisfaction, scores were high (average 7.8/10) for **This is Your Life** (103) and for **Ching Ching Story** (104), suggesting that participants were confident in their acquisition of mental health knowledge. Study adherence rates were reported for **Stigma-Stop**, 76.4% (122) and **Ching Ching Story**, 53.7% (104).

Regarding Engagement (see Table 2), no stakeholder involvement was reported on game development for the three adult games (RQ3), although two of them, **This is Your Life** (103) and the **Ching Ching Story** (104) had user satisfaction ratings, while **Stigma-Stop** (122) reported gameplay only (RQ4). Involvement scores were 3 for **This is Your Life** and the **Ching Ching Story**, and 1 for **Stigma-Stop** (122) (see Supplementary Table 1, Appendix 3 in Supplementary Material). Concerning Ethical Practices (RQ5), no findings emerged beyond descriptions of standard ethical procedures.

#### Step 1: At-Risk Groups/Suspected Mental Health Problems

Step one included five games, three for youth and two for young adults, described in six of the 49 (12.2%) studies. Youth games included a therapeutic game, **SPARX** (75, 79) for depression risk, an educational game, **POD Adventures** (97), for self-identified mental health needs and **GameSquad** (111), a series of exergames with coaching to increase physical activity and counter obesity among youth with co-occurring mental health problems. The two young adult games were an educational/mental health prevention game, **Reach Out Central** (107) for alcohol use, and the **Shots Game** (121) for alcohol use.

##### Youth Games

Regarding Processes (RQ1), three youth studies used qualitative methods (75, 79, 97), while the fourth was an RCT (111). In one **SPARX** (75) study, participants received information and a 5-min trailer on **SPARX**, but did not play the game. Regarding barriers to implementation, a game design issue concerning limited game selection in **GameSquad** (111) was viewed as a possible explanation for drop-off from gameplay over time. Two **SPARX** studies (75, 97) reported barriers to implementation concerning equity issues around a lack of local internet infrastructure that reduced access to digital games. Resources were also lacking for promoting **SPARX** with indigenous (Maori) families (79). Maori designs with appeal for indigenous youth and families were identified as a facilitator for reducing cultural barriers and providing support (79). Remote telehealth counseling was viewed in **GameSquad** (111) as a facilitator for program engagement.

Outcomes (RQ2) in terms of Impact and Effectiveness were largely anecdotal for this group of studies, except for **GameSquad** (111), an RCT that showed significant improvement in physical activity among participants. User satisfaction reflected positive reactions to the games, while recommendations highlighted the importance of personalization and reflected user preferences for home use of games (75). **SPARX** showed good face validity, effectiveness, and cultural relevance (79). Program adherence rates were not reported for any youth studies in Step 1. **GameSquad** (111) showed evidence of Sustainability, with 67% of participants reporting intentions to continue using the program. Regarding Equity, three youth studies were geared toward the needs of youth in rural, underserved areas of Australia (75) and New Zealand (79) (**SPARX**), while **POD Adventures** focused on help-seeking among students attending under-resourced schools in rural India (97). Both games included culturally relevant characters with different genders and body shapes, and representations of different social classes and languages. Translations of **POD Adventures** are available in English, Hindi and Konkani, a local language in Goa. A rating scale with smiley faces was used to assist comprehension.

On Engagement (see Table 2), stakeholder participation by youth, family, service providers and outside experts was considerable in Step 1 studies. Concerning game development (RQ3), youth, service providers and Maori, including a Maori game co-creator, Maori computer company and cultural experts, contributed to the design and development of **SPARX** (79) and co-design workshops that included youth were held. Youth and service provider participation in developing the **POD Adventures** (97) app was also provided through co-design workshops. Concerning game assessment (RQ4), youth and family provided evaluation and feedback for **SPARX** (75, 79), while service providers participated in focus groups for **POD Adventures** (97) where they expressed positive reactions to the narrative format, use of quizzes and rewards, game interactivity and real-life relatability. Testing sessions by youth for **POD Adventures** were also held, and both games included gameplay. Participation was also very robust for **GameSquad** (111), where parents served on an advisory team responsible for adapting the intervention (RQ3), in screening study participants as well as participating in gameplay and in coaching sessions with their children (RQ4). A youth exit questionnaire with game assessment was also offered. Involvement ratings in these youth studies were the highest in the review for **POD Adventures** and **SPARX**, which both scored 10, a study on Rainbow **SPARX** scoring 7, while **GameSquad** scored 3 on user engagement.

Enhanced Ethical Practices (RQ5) were reported for **POD Adventures** (97) and **GameSquad** (111). **POD Adventures** included a risk assessment question for low mood and a plain-language privacy/confidentiality statement for youth participants, while in **GameSquad** researchers withheld demographic data to protect against inferred identification of the 23 participants with a host of diagnoses.

##### Young Adult Games

Processes used in the two young adult games (RQ1) included a randomized clinical trial (121) and a pre-post game assessment (107). Implementation barriers concerned a game design issue affecting the **Shots Game** (121), in which limited game elements, personalization and a somewhat simplistic storyline resulted in a disappointing game experience. A similar critique about a storyline that did not sustain interest, particularly for males, was directed at **Reach Out Central** (107), and downloading requirements were also reportedly complicated. Study design/research issues included leaving game “dosage” to player discretion, resulting in a single playthrough for most participants and a shortened intervention period, both of which may have undermined results in **Reach Out Central**. Authors also expressed reservations about their decision to eliminate the 16-18-year age group, thereby avoiding the requirement for parental consent, after realizing that **Reach Out Central** had more appeal for younger players.

In terms of Impact (RQ2), players of **Reach Out Central** (107) showed positive improvement on alcohol use, psychological distress, and coping. Mental health literacy also improved for both sexes. Program satisfaction reached 90% in **Reach Out Central** (107), whereas in the case of the **Shots Game** (121), motivation to change (drink less) decreased among players using gamified visual probe training (VPT-G) while increasing among players of the non-gamified version (VTP-R) and the placebo (VPT-P) conditions. Adherence rates were 93.9% for the **Shots Game** (121) and 57.9% in **Reach Out Central** (107).

Regarding Engagement (see Table 2), no findings were reported related to stakeholder participation in game development for the young adult games (RQ3). On game assessment (RQ4), **Reach Out Central** (107) rated youth satisfaction and enjoyment, and both games were played by youth. Involvement ratings for these two studies were **Reach Out Central**: 3 and the **Shots Game**: 1, for gameplay only. No findings emerged on Equity or Ethical Practices (RQ5) in the young adult studies.

#### Steps 2-3: Mild to Moderate Mental Health Conditions

This largest category of games for mild to moderate mental health conditions included 16 games evaluated in 24/49 studies (49.0%). Five of the eight youth games were therapeutic games: **SPARX** (76–78, 80–83) for depression or depression/anxiety, **SmartCAT** (89, 90) for separation, social and general anxiety, **Dojo** (84) for anxiety/emotion regulation, **Minecraft** (120) for ADHD and **What Happened?!** (98) for binge drinking/alcohol use disorder, while two therapeutic games were labeled as adjunct to therapy: **Maya** (101) for depressive symptomatology and **Pesky gNATs** (91, 92) [originally called gNATs Island (91)], for low mood, depression or anxiety. There was one educational game, **The Journey** (108), for depression. The eight games for young adults included six therapeutic games: **On Track>The Game** (105) for first episode psychosis, **PlayMancer** (116) for bulimia nervosa, **RAW HAND** (117) for OCD, **Walk in My Shoes** (119) for PTSD, **e-SMART-MH** (106) for depression and **OCfree** (110) for OCD. One treatment facilitation game, the **Cockroach Game** (114), addressed specific phobia and a commercial game, **Journey** (109), depressive symptomatology.

##### Youth Games

Processes (RQ1) for the 16 youth studies included: seven RCTs (76–78, 83, 84, 98, 108), two open trials (81, 89), one pre-post game assessment (90), two qualitative studies (80, 101), one multiple case study (91), one mixed-method study (92) one study with sequential recall testing (120) and one secondary data analysis (82). Barriers to implementation reflected game design issues, including unspecified technical problems in **The Journey** (108), lack of personalization in **Pesky gNATs** (91) and a suspected lack of cultural fit for **SPARX** (76), delivered in a Dutch intervention study. Study design/research issues included inadequate sample size and high dropout (76), intervention/control games with similar aims that contributed to weaker results (84) and lack of time/resources to include parent participation (80). Regarding staff issues, some therapists reported fears of being “sidelined” by high client interest in the game intervention (91). Offering interventions on class time was a facilitator (76), as were opportunities for game playing alongside therapists in support of therapeutic elements (92).

Study outcomes (impact, effectiveness, sustainability, and equity: RQ2) included, in terms of Impact, significant improvement on key variables in four of the nine games: on depressive symptoms in **SPARX** (77, 83), depression in **The Journey** (108), lower anxiety in **SmartCAT** (89, 90) and memory consolidation in novel environments for ADHD in **Minecraft** (120). Effectiveness was further demonstrated for **SPARX** (83), **What Happened?!** (98) and for **SmartCAT** (90), which demonstrated significantly higher effectiveness in the gamified vs. non-gamified version of the app (89). On secondary measures, results were equal or better for **SPARX** vs. treatment as usual on quality of life measures, and in results of the Mood and Feelings scale and Enjoyment and Satisfaction scale (77). User satisfaction scores were high and commentary positive for six games, including **SPARX** (76–78, 83) and the Rainbow **SPARX** adaptation for LGB youth (81). The game designs and characters in **SPARX** enhanced Maori cultural identity, quality of life and hope (80). Among youth who played **The Journey** (108), 89% liked the game and would recommend it, while similar reactions occurred for users of **Pesky gNATs** (91). In addition to a 97% satisfaction rating by parents on **SmartCAT** (90), therapists also weighed in, giving **SmartCAT** an A+ rating on usability of the clinician portal, and praising **Pesky gNATs** for promoting therapeutic relationships and helping to transmit CBT concepts (91). One third of therapists endorsed **Maya** (101), evaluating the game as a sound treatment approach for externalizing emotions and preventing depression as well as a good fit with their therapeutic work. Therapists using **Pesky gNATs** (92) stated that the children enjoyed the game and responded well to exercises introduced by the characters. Program adherence was reported for 11 studies on the following six games: **Minecraft**, 94.4% (120); **The Journey**, 94% (108); **Dojo**, 93.5% (84); **SmartCAT**, 88.2% (90) and 85.7% (89); **SPARX**, 76.4% (78), 70.4% (81), 60% (77) and 30% (76), although adherence decreased gradually to 0% in **What Happened?!** (98), despite multiple reminders to keep players struggling with binge drinking engaged. Lucassen et al. (82) reported average completion of 4+ modules (“adequate dose”) for only 6% of intersex participants in a 5-year review of **SPARX** data.

Sustainability (RQ2) was positive in three of the five studies with measurable impact. Remission from depression persisted at follow-up for **SPARX** (77, 83); 30-day binge drinking was lower at 4-month follow-up for **What Happened?!** (98) and symptom improvement persisted at 2-month post-treatment for **SmartCAT** (90). Equity issues were addressed in **SPARX**, through game adaptations for sexual minority youth (81, 82), indigenous Maori youth in New Zealand (80) and socio-economically disadvantaged youth in rural areas of New Zealand and Australia (76, 83). Researchers also provided equity testing for internet access in **What Happened?!** (98).

Results on Engagement (see Table 2) reflected considerable stakeholder participation. Youth involvement in game development (RQ3) occurred for **SPARX** (77), **What Happened?!** (98), and for youth in conjunction with therapists in **SmartCAT** (89). Service providers contributed to game development for **SPARX** (81), while a range of outside experts in psychiatry collaborated in game development and approval of **Maya** (101). Parents were involved in game assessment (RQ4) for **What Happened?!**, and in two **SPARX** studies (76, 83), while service providers assisted with study recruitment for a **Dojo** study (84). In-study assessments of youth satisfaction or enjoyment were conducted for **Pesky gNATs** (91), **SPARX** (76–78, 80, 81, 83), **Maya** (101), **The Journey** (108) and **SmartCAT** (89), while therapists completed in-study game assessments for **Pesky gNATs** (91, 92) and **Maya** (101), and parents did the same for **SmartCAT** (90). All studies involved youth in gameplay, with the exception of one **SPARX** study with indigenous Maori participants (79). Involvement ratings for games in this group of studies were: **SPARX** (7 studies, with scores of 10, 7, four studies scored 3, and one study scored 1); **SmartCAT** (2 studies, scored 7 and 3, respectively); **What Happened?!**: 5; the following games had scores of 3: **Maya, The Journey, Dojo** and one of the two studies on **Pesky gNATs**; while the other scored 1, as did **Minecraft**.

Regarding Ethical Practices (RQ5), four studies, three of them **SPARX** interventions (76, 77, 81) and the other for **The Journey** (108), implemented safety measures and protections in the form of mood-monitoring questions, safety checks and referrals for high depression/anxiety or risk of self-harm, while two studies involving **Dojo** (84) and **What Happened?!** (98) provided written assurance that participant data would not be shared. In a **SPARX** trial, all students in one setting played the game so that study participants could not be singled out (83).

##### Young Adult Games

Processes (RQ1) used in these eight studies included two RCTs (106, 109), one clinical trial (110), two pre-post game assessments (117, 119), one mixed method study (105) and two single case studies (114, 116). The one barrier to study implementation involved a study design/research issue around burdensome travel requirements to the study site that affected recruitment and study feasibility (106).

Outcomes on young adult games for mild-moderate conditions (RQ2) included Impact, with five interventions showing significant improvement on their main variables: attitudes toward recovery in **On Track>The Game** (105), game feasibility (vs. controls) in **e-SMART-MH** (106), less binge eating in **PlayMancer** (116), on reaction scores and video game self-efficacy and attitudes toward the game for **Walk in My Shoes** (119) and on fear/avoidance in the **Cockroach Game** (114) for which Effectiveness was also demonstrated. The mobile CBT intervention, **OCfree** (110), demonstrated equal effectiveness as offline CBT for treating OCD, whereas the commercial game, **Journey** (109), had no effect on depressive symptoms, which persisted for 50.2% of players at follow-up. No results on secondary variables were reported. User satisfaction scores were 3.5/5 for **OCfree**, with 70% of participants recommending the game. The **Cockroach Game** (114) and **On Track>The Game** (105) for first episode psychosis were rated as very helpful, the latter praised for enhancing recovery, hope and treatment confidence, and for game interactivity and customization. Design elements eliciting user dissatisfaction were lack of self-tailoring in **e-SMART-MH** (106). Adherence rates were reported for two games only: **OCfree**, 91.4% (110) and **e-SMART-MH**, 46.7% (106). The single patient using the **Cockroach Game** was able to play daily with decreasing anxiety (114). Regarding Sustainability, two single case studies, the **Cockroach Game** (114) and **PlayMancer** (116), showed that gains were maintained or improved at 12-month follow-up. Two other studies noted a willingness of participants to continue using digital games post-trial (106, 110). Regarding Equity, two US studies prioritized recruitment of ethnic minorities (African American, Hispanic, mixed-race individuals) (105, 106).

Regarding Engagement (see Table 2), no young adult studies at Steps 2-3 reported on stakeholder involvement in game development (RQ3), whereas four studies reported on in-study user satisfaction, acceptability or other game assessments (RQ 4): **On Track>The Game** (105), **e-SMART-MH** (106), **OCfree** (110), and **Journey** (109). There was no participation in the other four young adult studies, but gameplay was recorded for the **Cockroach Game** (114), **PlayMancer** (116), **RAW HAND** (117), and **Walk in My Shoes** (119). Based on the criteria given in Table 2, involvement ratings for five of these studies were evaluated as 3: **On Track>The Game, e-SMART-MH**, **Dojo**, **Journey**, and **OCfree**, while the remaining four studies counted gameplay but no participation and were each rated 1. Only one study commented on Ethical Practices (RQ5). Virtual reality exposure to phobia, as described in the **Cockroach Game**, was considered more ethical than *in vivo* exposure which purposefully evokes distress in patients (114).

#### Step 4: Severe and Complex Mental Health Conditions

##### Youth Games

Seven games (9/49 studies: 18.4%) were played by youth with severe mental illness in residential institutions or inpatient/outpatient psychiatry units. They included four therapeutic games: **The SIMS Life Stories**^**TM**^ (113) for trauma, **SPARX** (74) for depression, **Muse** (112) for trauma and **RAGE-Control** for anger management (102); two relapse prevention games: **Arise** (99) for substance use disorder and **Recovery Warrior** (87, 88) for substance use disorder including opioid or marijuana use disorder, and a biofeedback game, **Dojo** (85, 86), that was used as adjunct to therapy.

Processes (RQ1) involved a full range of methodologies: three RCTs (86, 87, 112), one open trial (74), two pre-post game assessments (85, 88), one pilot survey (99), one qualitative study (113), and one single case study (102). Barriers to implementation included study design/research issues, e.g., lack of time and space in research settings, competing house/ward activities and illness acuity among participants (74, 113). Recruitment of patients with addiction was especially challenging due to a short time frame that did not allow for rolling enrollment and the inability to reach out directly to a small subpopulation of potential participants. They had to be recruited through providers, whose support was crucial, due to the sensitive nature of data being collected. Participants were later contacted through anonymous email accounts and phone (99). Implementation issues also emerged, as time constraints precluded some residential staff from involvement in research (113).

Study outcomes (RQ2) included game impact, effectiveness, sustainability, and equity. On Impact, significant positive associations were identified on key variables for three of the seven games: abstinence from drugs in **Recovery Warrior** (88), anxiety and externalizing behavior in **Dojo** (86) and post-traumatic symptoms and stress, anxiety, aggression and depression in **Muse** (112). Effectiveness was demonstrated for **Dojo** and **Muse** in youth residential settings (85, 86, 112), as well as strong acceptability ratings by youth and providers for **SPARX** (74) and **Arise** (99); strong four-week, self-efficacy scores on resistance to marijuana and 44.4% abstinence with **Recovery Warrior** (88), and lower pre-post scores on anxiety and aggressive behavior with **Dojo** (85, 86). On user satisfaction, acceptability and enjoyment, also part of Effectiveness, all nine studies reported positive results: social workers viewed **The SIMS Life Stories**^**TM**^ (113) as a good tool for engaging residents in therapeutic work and building therapeutic relationships, while several games were viewed by players as especially useful or helpful: **SPARX** (74), **RAGE-Control** (102), **Arise** (99), and **Dojo** (85, 86). **Muse** was enjoyed more and had a stronger treatment effect for male vs. female players (112). Moreover, study adherence, measured as program completion rates, was reported for seven studies that tested six games, as follows: **Arise**, 100% (99); **Dojo**, 90% (86), **Recovery Warrior**, 80% (87) and 66.7% (88); **Muse**, 77.7% (112), **The SIMS Life Stories**^**TM**^, 36.7% (113) and **SPARX** 10.0% (74). Regarding Sustainability, of the studies showing positive results, gains were not sustained at follow-up for **Recovery Warrior** (87), **Muse** (112) or for one study with **Dojo** (86). Equity, the final outcome measure, was largely neglected in Step 4 studies, although one study noted the need to personalize videogames for race, different socioeconomic contexts and literacy issues (99).

Regarding Engagement data for Step 4 (see Table 2), neither youth nor family involvement in game development occurred, but a five-member consultation board of addiction experts evaluated and revised an early prototype of **Arise** (99) (RQ3). On game evaluation (RQ4), residential social workers who delivered the **The SIMS Life Stories**™ (113) intervention completed semi-structured interviews, while **Arise** (99) included service provider and youth satisfaction ratings, the latter also rated for several other games: **RAGE-Control** (102), **Dojo** (85, 86), **Muse** (112), and **SPARX** (74). Ratings of 3 for youth involvement were calculated for six of the seven games, as follows: **Arise**, **The SIMS Life Stories**^**TM**^, **RAGE-Control**, **Muse**, **SPARX** and both studies on **Dojo; Recovery Warrior** scored 1.

Finally, on Ethical Practices, potential harms related to gaming disorder (RQ5) and mitigation strategies were not specifically addressed in Step 4 studies. However, rules against playing games without a defined mental health benefit in one institution for youth with complex trauma seemed to reflect this concern, obliging researchers to provide both TAU and **Muse** (112) to the intervention group. Study ethics protocols did include some important protections for vulnerable participants, however, like restricting data-sharing with parents and clinicians (99), private data storage and reinstatement of trauma therapy for vulnerable youth prior to the follow-up period (112). Authors of the **RAGE-Control** intervention cited an opinion that digital game interventions could potentially substitute for seclusion practices or use of restraints to control aggressive behavior (102).

##### Young Adult Games

There were no games for young adults at Step 4.

### Quality Assessment

All studies in the review were primary studies, representing substantial methodological variation (e.g., RCTs and clinical trials, pre-post game assessments, mixed methods, qualitative studies, and a number of single studies using other methods). The quality assessment was limited to RCTs, of which five were rated as “fair” (77, 78, 108, 109, 111) and the remainder “poor.” Methodological issues included lack of blinding of participants and outcome assessors, lack of treatment allocation concealment, small sample sizes, low adherence, loss to follow-up and lack of avoidance of other treatments. For most studies, the randomization methods used, power calculations, reasons for loss to follow-up and low adherence were underreported (see Quality Rating Table, Appendix 4 in Supplementary Material).

## Discussion

This “gaming my way to recovery” review aimed to identify the existing literature on digital game interventions targeting young people with a full range of mental health and substance use problems. According to an international 2021 review on digital games (123), game-based interventions exist for most of the disorders identified in psychiatry, but in various experimental stages. We identified and undertook an exhaustive review of 49 studies concerned with 32 relevant games and, using the new Stepped Care Model for Videogame Interventions, mapped the evidence on the overall aims of game interventions, how they have been implemented, evaluated, and supported through stakeholder participation, and whether using gamified interventions in youth mental health services revealed any harms.

The distribution of the 49 studies across the Model reveals that nearly half, 24 studies (49.0%) evaluated games for mild-moderate mental health problems (Steps 2-3: 16 games), followed by 10 studies (20.4%) for games promoting mental health wellness and education among undiagnosed youth (Step 0: 7 games); nine studies (18.4%) on youth games for severe mental illness or substance use disorder (Step 4: 7 games) and six studies (12.2%) for at-risk groups or suspected mental health problems (Step 1: 5 games). Recent international interest in developing digital games for youth is reflected in the broad geographic scope of this literature across 12 countries. Games are being developed to address rising rates of mental health problems among young people, particularly since the pandemic (7, 10, 124–126) as well as low service use (17, 18). Nearly twice as many games in this review targeted youth, compared with games for young adults (65.7 vs. 34.3%). Authors of 11 games made specific efforts to promote equity and inclusiveness, most focusing on ethnic, racial, and sexual minority youth and those living poor, rural, or underserved areas who receive little help or mental health treatment. Such efforts may extend the reach of mental health interventions, while enhancing the “*appealing potential*” of videogames as a treatment delivery vehicle (34). As noted, **SPARX** was by far the most studied (10 studies) and disseminated intervention, reaching three countries beyond New Zealand, as well as the only game implemented across all levels of illness severity (Steps 1, 2-3, and 4).

### Evidence for the Capacity of Digital Games to Support Youth Mental Health Services and Care

Digital game interventions are effective and may have added value for youth followed in mental health services. The overall strength of the evidence in this review is suggested by the number of studies with measurable outcomes (*n* = 38: 77.6%). They include 18 (36.7%) randomized studies with control/comparison groups (13 RCTs, four randomized clinical trials and a between-groups experimental design) and 20 (40.8%) uncontrolled quantitative or mixed method studies (11 pre-post game assessments, three open trials, one structural equation model, one mixed method study, two pilot surveys and two studies using sequential recall testing). The remaining 11 studies (22.4%), consisting of six qualitative studies, three single case and one multiple case study, and a five-year, secondary data analysis, amplified the findings especially in terms of user satisfaction and acceptability and in describing barriers and facilitators related to implementation of the games.

Regarding specific outcomes, two-thirds of the 38 quantitative studies reported significant improvement on one or more key variables, and 12 studies described effectiveness on some or all comparisons. Further positive results emerged on secondary variables for **SPARX** (Steps 2-3) on quality of life, life satisfaction and mood, and for **Aislados** (Step 0) on improved affect and mental health among participants. Regarding sustainability, the reported gains carried over to the follow-up period in eight studies: **Arise**, **The SIMS Life Stories**^**TM**^, and **Dojo** (Step 4) and for **SPARX**, **What Happened?!**, **SmartCAT**, **PlayMancer** and the **Cockroach Game** (Steps 2-3). As well, 18 studies (36.7%) measured or described user satisfaction, with better than average ratings on all studies and positive results from the qualitative data. Program adherence, another possible indicator of user satisfaction, was reported for 28/49 (57.1%) of studies in the review. While previous research reported low study adherence for digital mental health interventions (29, 127–129), average study retention for videogame interventions in this review was 64.5%. Our results compared favorably with a meta-analysis on adherence in gamified interventions for 61 interventions reported as 54.0% (SD: 24.6%) and where considerable variation was identified based on a number of specific gaming features (130).

Another promising aspect of these 32 games for possible adaptation to mental health settings and further testing was that nearly all were serious games. Steps 1-4 for young people with mental illness or symptomatology included 15 therapeutic games and two described as treatment facilitation games, two relapse prevention games, one cognitive retraining game, five games specifically developed as adjunct to therapy including four using biofeedback, and three educational games. Previous research suggested that the game environment itself may promote engagement, as players have access to a stimulating learning environment that facilitates knowledge acquisition, behavior change through repeated rehearsal of new behaviors, emotional control, and social learning (131–133). Moreover, the addition of a Step 0 in the new Stepped Care Model for Videogame Interventions recognizes the contribution of studies geared toward mental wellness and education for undiagnosed young people at large, with 10 studies carried out in schools or university settings, or at home.

Unfortunately, not all videogame interventions offered a rich and interactive game experience, which seems to have impacted on the effective implementation of certain interventions. Game-related issues ranged from disappointing game experiences due to limited game elements, storylines or game menus and lack of personalization or cultural fit, to the quality of therapeutic relationships or lack of therapist support (i.e., self-help formats), to technical glitches. Such problems may have provoked drop-off from gameplay or affected study results. Barriers to implementation also included issues around study design/research and equity, the former related to recruitment of hard-to-reach participants, e.g., those who used drugs, insufficient doses of the game, or active control groups with aims comparable to those of the intervention, which weakened study results. When intensity of gameplay (number of sessions) was left to player discretion in one study (107), most played the game only once providing insufficient data for analysis. Other barriers concerned staff (Step 4) for whom time constraints prevented active involvement in research, and certain therapists who feared being “sidelined” by patient interest in videogames (91). Finally, material resources were sometimes lacking, such as local internet infrastructure in remote or poor areas, limiting game use.

The identified barriers in these studies suggest possible improvements for building game capacity. Use of the Collaboration on Maximizing the impact of E-therapy and Serious Gaming (COMETS) framework (132) could help improve videogame interventions, the player's experience and overall impact of the mental health intervention in naturalistic settings. The COMETS framework is based on four pillars for change: (1) user-centered approaches, including both user-centered program designs and individualization within programs; (2) engagement of the player using processes such as gaming and gamification; (3) active collaboration in program development, testing, and data sharing across both the mental health and industry sectors in order to achieve higher quality, more sustainable outcomes with greater reach and decreased costs of developing interventions to be shared across sectors; and (4) rapid testing and implementation, including rapid research designs and implementation planning as well as measurements for reach, engagement, effectiveness and timeliness of game implementation, to ensure that interventions ready for use will retain their appeal.

### Engagement and Stakeholder Participation

The Engagement construct in the Stepped Care Model for Videogame Interventions brought together results for participation by youth, family, and service providers, as well as outside experts who were involved in various aspects of game development, evaluation, study implementation and testing. Studies in the review presented these results both in narrative form and as measurable results, leading to the development of Table 2, which synthesizes these findings across four levels, based on the adapted *Ladder of Children's Participation* (70). While engagement by all stakeholder groups is described in Table 2, the “involvement” score calculated for each game was based on the cumulative scores for youth engagement only, recognizing their predominant role as primary end-users of the games. While gameplay may not be viewed as “engagement,” the scores for gameplay by youth in the context of study participation were included as a key element of their involvement. In the seven studies reporting on the four highest scoring games: **SPARX**, P**OD Adventures**, **SmartCAT**, and **What Happened?!**, the elevated scores on youth participation reflected greater inclusion of youth in game design and development (score: 4) and in consultation activities (score: 3). Four of these studies tested the **SPARX** intervention, revealing scale-up of the game through adaptations for indigenous Maori, sexual minority, and socioeconomically deprived youth, as well as diffusion of the game beyond New Zealand. The next group of 25 studies evaluated 17 new games (8 repeats) with a score of 3, as these games were informed by data on youth experience, acceptability, or user satisfaction. Finally, 17 studies that tested 11 games (six repeats) found no evidence of youth participation, but were rated 1 each for gameplay (see also Study Data file, Appendix 2 in Supplementary Material).

With nearly one third of games in the review (see Table 2 for full results) showing no evidence of any youth participation and only four with intense engagement by youth in initial stages of game design and development, awareness about the importance of youth participation in videogame creation is clearly lacking. Research on therapeutic games in mental health has shown that involving young service users early in the design process through participatory and user-centered approaches produces better game mechanics and enhances user engagement in both games and the treatments they support (45). As well, several frameworks and models are available to guide and monitor patient engagement in treatment and research (134–138). Promoting patient engagement in treatment is essential, helping them to develop greater awareness of their mental health conditions, activate self-management behaviors and gain greater control over their treatment and recovery journeys. Moreover, patient engagement in research, including videogame implementation studies, promotes the development of better interventions tailored to the needs of service users.

### Potential Harms and Benefits of Digital Game Interventions

All the articles reviewed met standard ethical requirements and were screened by institutional research ethics boards. More than 25% featured enhanced ethical practices, including safeguards against risks of high depression/anxiety or self-harm. Other benefits included providing written assurance to youth participants that their data would not be shared or inviting all students in school settings to participate in research so that study participants with depressive symptoms would not be singled out. One study noted that digital games could substitute for use of seclusion and restraints to control aggressive behavior (102), while another promoted virtual reality exposure to specific phobia as less distressing and more ethical than *in vivo* exposure (114).

Remarkably, the literature under review was almost completely silent on the pivotal question of whether digital game interventions may be associated with gaming disorder or related harms. In a qualitative study, one therapist cautioned against allowing youth already addicted to videogames to use videogame interventions in therapy (101), while another study described an injunction against gameplay with no defined mental health benefit in an institution for traumatized youth, seemingly to prevent overuse of games by residents (112). A partial explanation for the lack of attention to possible addiction in applied games for mental health may relate to their serious therapeutic purposes, as described above, such as symptom reduction, treatment facilitation or support, education or cognitive retraining. As well, digital games have been used effectively to treat symptoms in a variety of mental health conditions ranging from depression and anxiety, PTSD, autism spectrum disorder and ADHD to alcohol use disorder (34, 38, 139). Yet another explanation may be that digital games have become such an integral part of everyday life that therapists no longer consider them to be any more addictive than other daily activities and hobbies such as exercising, watching tv shows, reading, etc. Nor did the two commercial games in this review suggest a particular risk of overuse or addiction. **Journey** (109) showed no effectiveness in preventing depression and player engagement was low (109). The other, a therapeutic adaptation of a commercially available game, **The SIMS Life Stories**^**TM**^ (113), was viewed by residential social workers as a potentially useful tool in individual therapy with youth affected by complex trauma. As such, use of these commercial games, where user engagement in therapeutic work was low or challenging, suggested little, if any, risk of gaming addiction.

### Limitations

Several limitations of this study should be acknowledged. Regarding the research process, some relevant studies may have been overlooked, particularly non-English studies, despite a thorough database search and screening by two teams of two reviewers. Second, while the methodological procedures were thorough and results included mainly positive outcomes, some findings like those on study effectiveness were based mainly on descriptive rather than statistical data. User satisfaction, the other measure suggesting effectiveness and assessed with both quantitative scales and open questions, was high. Third, regarding study retention, it should be acknowledged that other factors may have affected program adherence or attrition in this review, including participant issues external to the research. Finally, and most important, were limitations associated with the stepped care framework. While the new Stepped Care Model for Videogame Interventions seeks to establish a comprehensive, rigorous, and consistent process for describing and evaluating studies, a lack of detail in the selected studies forced a merger of “mild” and “moderate” mental health conditions into a Step 2-3 and prevented us from adapting elements from a staging model in developing this model. Moreover, stepped care models are often based on intervention levels, not severity levels, in mental health and the criteria for “stepping up/down” are not clearly established. As well, a “Step 0,” or “no-diagnosis” level had to be created to account for studies focused on mental wellness and education. Moreover, data gathered from published articles does not provide a comprehensive view of the intervention, nor is the information provided by authors on their research process always complete. Studies in this review may have contained important data not captured by the model or inadvertently omitted, for example, information on levels of service, integrated services, or continuity of care. Not least, authors may possibly disagree with how data from their studies were categorized or interpreted.

What could be viewed potentially as exceptions to the study inclusion criteria should be explained: two studies using commercial games were included in the review, as they were both used for therapeutic purposes (one game for depression management and the other as adjunct to emotion regulation treatment); five youth studies were included even though the lower participant age limit was <12 because the mean ages met the inclusion criteria set for this study. The exceptions were two studies of the same game (**SmartCAT**) where the mean age (11.19) was slightly lower. For the other three cases the mean ages were within the prescribed age range of the review. Finally, the **GameSquad** study, a series of exergames promoting wellness among youth, was retained as more than half of participants had ADHD, and 26% had anxiety disorders and/or depression.

### Future Research Directions

This review provides an initial categorization of digital game interventions for youth and young adults appropriate to different levels of severity (Steps) and a broad range of diagnoses in mental health and substance use disorder. The benefits and limitations of various interventions for specific diagnoses need to be better understood and further testing is required. Future research should include a meta-analysis comparing the efficacy of digital game interventions as well as analyses of game effectiveness in relation to stakeholder engagement and other outcomes, based on well-controlled studies. Moreover, research needs to focus more on participation by young users to improve engagement with the games, and, crucially, on eliciting participation by service providers and family to promote the transformation of stand-alone digital game interventions into standard tools for mental health treatment in clinical and community settings (schools and home) for youth. Opportunities for blending digital and face-to-face treatment should also be explored (140). Potential harms in the therapeutic use of digital games need to be better understood and more research is needed to unpack the complexity of gaming disorder, deeply rooted in the relationship between the user and the digital technology, and to find a proper framework that promotes safety in the use of digital interventions, particularly videogame interventions (51). The evidence on safety and effectiveness of games destined for clinical practice needs to be clearly established and the persuasive components of games, both ethical and transparent, with full control given to the user. Frameworks like COMETS that prioritize patient engagement in treatment and research can offer valuable tools to overcome multiple barriers related to the uptake of digital game interventions.

## Data Availability Statement

The original contributions presented in the study are included in the article/Supplementary Material. Further inquiries can be directed to the corresponding author.

## Author Contributions

MF, JS, SM, SF, and SMHS completed screening and data extraction. MF, JS, and SM analyzed the data. MF and JS wrote the first draft of the manuscript. All authors designed the study. All authors contributed to the interpretation and subsequent edits of the manuscript.

## Funding

This study was supported by a combination of grants from Frayme, International Knowledge Translation Platform, Ottawa, Ontario, Canada, the Healthy Brains, Healthy Lives (HBHL) New Recruit Start-Up Supplements Award (MF), and the Fonds de Recherche du Québec–chercheurs-boursiers Junior 1 Award (grant no. 283375) (MF).

## Conflict of Interest

The authors declare that the research was conducted in the absence of any commercial or financial relationships that could be construed as a potential conflict of interest.

## Publisher's Note

All claims expressed in this article are solely those of the authors and do not necessarily represent those of their affiliated organizations, or those of the publisher, the editors and the reviewers. Any product that may be evaluated in this article, or claim that may be made by its manufacturer, is not guaranteed or endorsed by the publisher.

## Abbreviations

ADHD: Attention-deficit/hyperactivity disorder
CBT: Cognitive behavioral therapy
OCD: Obsessive compulsive disorder
PRISMA: Preferred Reporting Items for Systematic Reviews
PTSD: Post-traumatic stress disorder
RCT: Randomized controlled trial
RQ: research question.
