# Supplementary material for: Gaming My Way to Recovery: A Systematic Scoping Review of Digital Game Interventions for Young People's Mental Health Treatment and Promotion
Source: Front Digit Health. 2022 Apr 7;4:814248. doi: 10.3389/fdgth.2022.814248 (PMC9021794; doi:10.3389/fdgth.2022.814248)
Supplement: Supplementary file 2 [file Data_Sheet_2.docx]

**Supplementary Table 1. Data for 49 studies using the Stepped Care Model for Videogame Interventions as a conceptual framework**

| **Population and mental health conditions** | **Focus and types of digital game interventions** | **State of knowledge: processes, impact, effectiveness, sustainability, equity, engagement, ethical practices** |
| --- | --- | --- |
|  | | |
| **Step 0: Mental health promotion/prevention/education and youth population-based interventions (7 games; 10/49 studies: 20.4%)** | | |
| **YOUTH:**  No diagnosis (Cangas, 2017; Cejudo, 2020; David, 2018, 2019; 2019a; 2021; Huen, 2016)  **YOUNG ADULTS:**  No diagnosis (Kelders, 2018; Li, 2013; Mullor, 2019) | **YOUTH**:  Wellness promotion or mental health prevention:  **Aislados** (Cejudo, 2020): Social and Emotional Learning  **REThink** (David, 2019, 2019a, 2021): Rational Emotive Behavior Therapy; Rational Emotive Behavior Education (REBT/ REBE)  **Professor Gooley and the Flame of Mind** (Huen, 2016): CBT; Positive Psychology, Interpersonal skills training  Mental health education:  **Stigma-Stop** (Cangas, 2017): NA  **Feeling Better** (David, 2018): Rational Emotive Behavior Therapy; Rational Emotive Behavior Coaching (REBT/ REBC)  **YOUNG ADULTS**:  Mental health prevention:  **This is Your Life** (Kelders, 2018): Self-determination Theory; Positive Psychology  Mental Health Education:  **Ching Ching Story** (Li, 2013): CBT; Diathesis-Stress Model of Depression  **Stigma-Stop** (Mullor, 2019): NA | **YOUTH**:  **Processes**: The seven studies on five games for youth included two randomized clinical trials (David, 2019, 2019a); three pre-post game assessments (Cangas, 2017; Cejudo, 2020; David, 2021); a pilot sequential game assessment (David, 2018); and one structural equation model (Huen, 2016). Barriers: game issues: duration of game and intensity of practice may have been insufficient to reach potential magnitude of change (David, 2021); high attrition rates possibly due to self-help format; need for therapist or administrative support (Huen, 2016); study design issues: small sample size and lack of control group in pilot (David, 2018).  **Impact**: Results showed significant improvement on main variables of interest reported for all five games on mental health promotion/prevention and education for youth: for **Stigma-Stop**, reduced stigma (Cangas, 2017); for **Aislados**, better health-related quality of life and mental health (Cejudo, 2020); less depressive mood in **Feeling Better** (David, 2018); improved functional emotion in **REThink** (David, 2019, 2019a, 2021) and user learning/psychological well-being in **Professor Gooley and the Flame of Mind** (Huen, 2016).  Secondary measures (e.g., life satisfaction, quality of life, other psychological functioning variables; perceived stigma, economic outcomes): more positive affect and better mental health (Cejudo, 2020).  **Effectiveness**: demonstrated for **REThink**: moderate effect size for emotional symptoms, large effect on depressive mood (David, 2019, 2021).  User satisfaction/acceptability: significantly better satisfaction scores at mid-intervention, favoring **REThink**, but no differences at post-intervention (David, 2019); 75% would recommend **Stigma-Stop**; high scores on usefulness of program (Cangas, 2017). Study retention was 88.0% (David, 2018); 86% (David 2019); 86% (David 2019a), 38.6% (Huen, 2016); NA for Cejudo, 2020; David, 2021; and Cangas, 2017.  **Sustainability**: Scant information on sustainability offered in two studies: results were not sustained after three trials for **Feeling Better** (David, 2018) and satisfaction with the game in the intervention group, versus controls, also faded at post-test for **REThink** (David, 2019).  **Equity:** All secondary students in Hong Kong invited to the Huen, 2016 study, no exclusion criteria.  **Engagement**: (See Table 3)  **Ethical practices**: NA.  **YOUNG ADULTS**:  **Processes**: The three studies with young adults included one between-groups experimental design (Kelders, 2018) and two pre-post game assessments (Li, 2013; Mullor, 2019). Barrier: study design: inability to measure cognitive and affective engagement may have been due to variables used (concepts lacking clear definition in the literature) (Kelders, 2018).  **Impact**: Results showed significant improvement on main variables of interest: more game involvement and flow in gamified vs. non-gamified condition reported for **This is Your Life** (Kelders, 2018); on mental health literacy in the **Ching Ching Story** (Li, 2013); and reduced stigma (similar between the game and face-to-face contact or a professional talk in **Stigma-Stop** (Mullor, 2019).  Secondary measures (e.g., life satisfaction, quality of life, other psychological functioning variables; perceived stigma, economic outcomes): NA.  **Effectiveness**: Results described the gamified version of **This is Your Life** (Kelders, 2018) as more effective than a non-gamified version of the game on cognitive and affective engagement, but not on behavioral or affective engagement alone. The **Ching Ching Story** (Li, 2013) was most effective in enhancing mental health literacy. **Stigma-Stop** (Mullor, 2019) was found to have similar effectiveness in reducing stigma compared with face-to-face contact, satisfying H1, and compared with a talk delivered by a professional; H2, stating that the game would be more effective than the talk, was not supported. User satisfaction: Participants valued intervention highly, with an average rating of 7.8/10 for **This is Your Life** (Kelders, 2018); the **Ching Ching Story** showed participant confidence in acquired mental health knowledge (Li, 2013); and NA on **Stigma-Stop**. Adherence was 76.4% (Mullor, 2019) and 53.7% (Li, 2013), NA for Kelders, 2018.  **Sustainability**: NA  **Equity**: NA  **Engagement** (See Table 3)  **Ethical Practices**: NA |
| **Step 1: At-risk groups/suspected mental health problems (5 games; 6/49 studies: 12.2%)** | | |
| **YOUTH:**  Depression (at risk) (Shepherd, 2015; Cheek, 2014)  Self-identified mental health needs (Gonsalves, 2019)  Obesity (co-occurring with psychiatric conditions): (Bowling, 2021)  **YOUNG ADULTS**:  Alcohol use: (Boendermaker, 2016; Shandley, 2010) | **YOUTH:**  Therapeutic interventions:  **SPARX** (Shepherd, 2015; Cheek, 2014): CBT  Educational/problem solving game:  **POD Adventures** (Gonsalves, 2019): Stress Coping Theory  Exergames with coaching:  **GameSquad** (Bowling, 2021): Social Cognitive Theory, Reserve Capacity Model; Family Ecological Model.  **YOUNG ADULTS**:  Educational game: mental health prevention:  **Reach Out Central** (Shandley, 2010): CBT  Cognitive Retraining game:  **Shots Game** (Boendermaker, 2016): Cognitive bias modification of attention. | **YOUTH**  **Processes**: Three youth studies were qualitative (Shepherd, 2015; Cheek, 2014; Gonsalves, 2019) and one RCT (Bowling, 2021). In Shepherd, 2015, Maori participants did not actually play **SPARX** but received an information session with a five-minute trailer and PowerPoint demonstration of the game. Barriers: Game issues: limited menu of exergames may have affected drop-off from gameplay in later weeks (Bowling, 2021); Equity issues: lack of local internet infrastructure in poor, or remote areas, limiting private use of the game (Cheek, 2014; Gonsalves, 2019); family resources needed to encourage uptake among the Maori (Shepherd, 2015); Facilitators: designs that appeal to indigenous youth/families may reduce barriers and support game uptake (Shepherd, 2015); remotely delivered counseling via telehealth reduced barriers to participation, met psychosocial needs independently of game and encouraged continued engagement (Bowling, 2021).  **Impact**: Reports on impact were anecdotal for all studies, reflecting positive reactions to the games, e.g., importance of personalization, choice of avatar; user preferences for home use (Cheek, 2014), while **GameSquad** (Bowling, 2021), the RCT, showed improvement in exercise among participants.  Secondary measures (e.g., life satisfaction, quality of life, other psychological functioning variables; perceived stigma, economic outcomes): NA.  **Effectiveness**: User satisfaction/acceptability: **SPARX** had good face validity, was potentially appealing and effective; cultural relevance viewed as important for engaging Maori; whanau important for well-being of youth (Shepherd, 2015). Adherence rates were not reported for any of the youth studies.  **Sustainability**: An exit survey in Bowling, 2021 found that 67% of participants intended to continue physical activity using **GameSquad.**  **Equity**: Three youth studies were geared toward needs of youth in rural, underserved areas, two studies on **SPARX** and the **POD Adventures** game, tested with help-seeking students in low-resource schools in India (Gonsalves, 2019). **SPARX** was introduced to Maori youth and investigated for cultural relevance to support this population and help reduce barriers (Shepherd, 2015), and was also transferred to a remote, poorer area of Australia with limited local internet infrastructure (Cheek, 2014), somewhat like the setting described by Gonsalves, 2019 in rural India. **POD Adventures** also includes culturally relatable characters representing a mix of genders, body shapes, social classes, and names; particular attention to language (available in English, Hindi and Konkani, a local language in Goa) and adaptations for literacy issues (e.g., concrete, specific language, use of “smiley faces” to aide comprehension).  **Engagement** (see Table 3)  **Ethical practices**: One study with a small sample (n=23) reported withholding demographic data to protect participants from inferred identification (Bowling, 2021).  Gonsalves, 2016 incorporated a risk assessment question encouraging participants reporting low mood to approach a school counselor for help. This study also included a privacy and confidentiality statement drafted in simple language for youth participants.  **YOUNG ADULTS**:  **Processes:** One study was a pre-post game assessment (Shandley, 2010) and the other a clinical trial (Boendemaker, 2016). Barriers: Game issues: intervention with disappointing game experience against higher expectations due to limited game elements, no storyline and little personalization (Boendemaker, 2016); insufficiently complex storylines in **Reach Out Central** (Shandley, 2010) may have dampened interest or further game exploration; game less appealing to males. Authors also noted that the intervention was more geared toward younger youth. Technical issues: downloading requirements were complicated and time-consuming; work needed on game mechanics. Study design: Eliminating the 16-18-year age group to avoid parental consent requirement may have negatively affected results, according to authors. Short intervention period may have undermined effectiveness; since “dosage” was left open, most participants played through once, which was viewed as inadequate to obtain results (Shandley, 2010).  **Impact**: For **Reach Out Central** (Shandley, 2010), positive improvement on alcohol use, psychological distress and coping, resilience, and quality of life for females; non-significant worsening effect for males on help-seeking, avoidance, and resilience. Improvements on health literacy for both genders.  Secondary measures (e.g., life satisfaction, quality of life, other psychological functioning variables; perceived stigma, economic outcomes) NA.  **Effectiveness**: User satisfaction/acceptability: Program satisfaction was 90% in Shandley, 2010; regarding the **Shots Game,** participants were not satisfied with minimal game elements (reward system, fancy graphics). Adherence rate was 57.9% in Shandley, 2010; NA for Boendermaker, 2016.  **Sustainability: NA.**  **Equity**: little personalization in the study intervention (Boendermaker, 2016).  **Engagement**: (See Table 3)  **Ethical practices**: NA |
| **Steps 2-3: Mild to moderate mental health conditions (16 games; 24/49 studies: 49.0%)** | | |
| **YOUTH**:  Depression (Fleming, 2011; Lucassen, 2015, 2021; Merry, 2012; Poppelaars, 2016; Shepherd, 2018; Stasiak, 2014)  Depressive symptomatology (Carrasco, 2016)  Low mood/anxiety (van der Meulen, 2019)  Anxiety (Pramana, 2018; Scholten, 2016)  Depression/anxiety (Coyle, 2011; Kuosmanen, 2017)  Separation/social anxiety (Silk, 2020)  ADHD (Baumann, 2020)  Binge drinking- alcohol use disorder (Jander, 2016)  **YOUNG ADULTS**:  Depression (Pinto, 2015)  Depressive symptomatology (Poppelaars, 2021)  First episode psychosis (Olivet, 2019)  PTSD (Kreutzer, 2015)  OCD (Hong, 2018; Hwang, 2021)  Specific phobia (Botella, 2011)  Bulimia nervosa (Fagundo, 2014) | **YOUTH**:  Therapeutic games:  **SPARX** (Fleming, 2011; Kuosmanen, 2017; Lucassen, 2015; 2021; Merry, 2012; Poppelaars, 2016; Shepherd, 2018): CBT  **SmartCAT** (Pramana, 2018; Silk, 2020): CBT  **Dojo** (Scholten, 2016) Emotion regulation training; training heart variability via biofeedback  **Minecraft** (Baumann, 2020): Behavioral tagging; Synaptic tagging and Capture Theory  Complex health intervention framework  **What Happened?!** (Jander, 2016): I-Change Integrated Model  Therapeutic game (adjunct to treatment)  **Maya** (Carrasco, 2016): Cognitive behavioral and interpersonal theories; Psychoeducation  **Pesky gNATs** (formerly gNATs Island – NOTE: name changed with author’s permission) (Coyle, 2011; van der Meulen, 2019): CBT; Developmental Psychology; Learning Theory  Educational game:  **The Journey** (Stasiak, 2014): CBT; Psychoeducation  **YOUNG ADULTS**:  Therapeutic games:  **On Track>The Game** (Olivet, 2019): Self-determination Theory; Theory of Cognitive Apprenticeship  **PlayMancer** (Fagundo, 2014): Video game therapy; CBT  **RAW HAND^©^** (Hong, 2018): Exposure and Response Prevention Therapy  **Walk in My Shoes** (Kreutzer, 2015): Expectancy Theory; Technology Acceptance Model  **e-SMART-MH** (Pinto, 2015): Cognitive behavioral-structured communication strategy  **OCfree** (Hwang, 2021): CBT  Treatment facilitation:  **Cockroach Game** (Botella, 2011): Bio-information Theory of Emotion; Augmented Reality  Depressive symptoms (commercial game):  **Journey** (Poppelaars, 2021): NA | **YOUTH:**  **Processes**: Of 16 youth studies, there were seven RCTs: (Fleming, 2011; Jander, 2016; Kuosmanen, 2017; Merry, 2012; Poppelaars, 2016; Scholten, 2016; Stasiak, 2014), two open trials: (Lucassen, 2015; Pramana, 2018), one pre-post game assessment: (Silk, 2020), one mixed methods: (van der Meulen, 2019), two qualitative studies: (Shepherd, 2018; Carrasco, 2016); one multiple case study: (Coyle, 2011), one secondary data analysis: (Lucassen, 2021) and one study with sequential recall testing: (Baumann, 2020).  Barriers: game issues: Re **Maya**, used as adjunct to therapy, the nature of the therapeutic relationship may have influenced game experience, e.g., oppositional attitude toward therapist could negatively dispose player to game (Carrasco, 2016); game customization viewed unfavorably by therapists; serious games seen as less appropriate for entrenched “gamers” (van der Meulen, 2019); technical problems, resolved but negatively viewed by players (Stasiak, 2014); study design issues: results/game impact affected by inadequate sample size (+high dropout) (Kuosmanen, 2017); equal reduction in anxiety at follow-up likely due to active control with same aim as the intervention (Scholten, 2016); Staff issues: therapist fears of being “sidelined” by high client interest in game (Coyle, 2011). Equity issues: lack of personalization (Coyle, 2011); intervention that lacked cultural fit (Kuosmanen, 2017); no time/resources for parent involvement in study (Shepherd, 2018).  **Impact**: For RCTs, trials and pre-post evaluation: significant improvement on main variables for game intervention in six studies: on depressive symptoms in **SPARX** (Merry, 2012; Fleming, 2011), on depression in **The Journey** (Stasiak, 2014), lower anxiety in **SmartCAT** (Pramana, 2018), significant impaired memory consolidation versus controls after exploring familiar environment, but significantly better with novel environment in **Minecraft** (Baumann, 2020). Also, 66.67% no longer met DSM (Diagnostic and Statistical Manual of Mental Disorders) criteria for pre-post anxiety in **SmartCAT** (Silk, 2020).  Secondary measures (e.g., life satisfaction, quality of life, other psychological functioning variables; perceived stigma, economic outcomes): **SPARX** ≥ treatment as usual on quality of life, Enjoyment and Satisfaction Scale and on Mood and Feelings questionnaire (Merry, 2012).  **Effectiveness**: described/demonstrated for **SPARX** (Fleming, 2011), **What Happened?!** (completers only) (Jander, 2016) and in **SmartCAT** (post intervention increase to 86.2% on anxiety reduction at two-month follow-up) (Silk, 2020); app usage significantly higher for gamified versus non-gamified **SmartCAT** (Pramana, 2018); **Maya** (Carrasco, 2016) viewed as extension to therapy – a way to “take therapy home”; yet also concern that oppositional patient attitudes toward therapist could negatively dispose patients when playing the game.  User satisfaction/acceptability: **SPARX**: players found the game engaging (Fleming, 2011); player satisfaction score 6/10, re recognizing negative thoughts, more satisfying relationships, feeling better about self (Kuosmanen, 2017); high satisfaction with Rainbow **SPARX** as relevant and engaging (Lucassen, 2015); satisfaction with **SPARX** for learning at own pace, New Zealand “look and feel” and game design for youth (Merry, 2012); similar positive satisfaction with **SPARX** from controls (Poppelaars, 2016); **SPARX** improved quality of life, hope, enhanced Maori cultural identity: e.g., designs, characters; game easy to understand, enjoyable (Shepherd, 2018); **SmartCAT**: users satisfied with visual appearance, ease of use, good fit with daily routines and helpful when feeling anxious (Pramana, 2018); very high user/parent satisfaction (97%); high rating on usability; A+ therapist rating for usability of clinician portal (Silk, 2020); **Pesky gNATs**: most users found game extremely enjoyable and helpful and would recommend it; positive change (Coyle, 2011); **The Journey**: 89% of participants liked game and would recommend it (Stasiak, 2014). Therapist satisfaction: Therapists felt **Pesky gNATs** enhanced therapeutic relationship and helped transmit CBT concepts (Coyle, 2011); therapist acceptability reported as 1.88/7, with one third endorsing potential benefit of the **Maya** intervention (Carrasco, 2016); game offered sound treatment approach; fit with psychotherapeutic work; may help communication/connection with patients; content mirrors real life; helped patients externalize emotions; good tool for preventing depression. Therapists viewed **Pesky gNATs** (van der Meulen, 2019) as enjoyable for children, who generally liked the game and responded well to exercises introduced by game characters. Program adherence rates were 93.5% (Scholten, 2016), 94.4% (Baumann, 2020); 65.3% (Fleming, 2011); 70.4% (Lucassen, 2015); 60.0% (Merry, 2012); 76.4% (Poppelaars, 2016); 85.7% (Pramana, 2018); 88.2% (Silk, 2020); 94.0% (Stasiak, 2014); 30% (Kuosmanen, 2017); and 0% (Jander, 2016) despite multiple reminders to participants; NA: Coyle, 2011, and Shepherd, 2018. Lucassen, 2021 reported average completion of four+ modules (“adequate dose”) for only 6% of intersex participants in studies of **SPARX** over a five-year period.  **Sustainability**: For the five games with measurable impact or effectiveness (**SPARX**, **The Journey**, **SmartCAT**, **Minecraft** and **What Happened?!**), gains in depression remission with **SPARX** were maintained at follow-up (Merry, 2012; Fleming, 2011); significant decrease in 30-day binge drinking at four-month follow-up for players of **What Happened?!** (Jander, 2016) versus controls, while symptom improvements persisted two months post-treatment in **SmartCAT** (Silk, 2020).  **Equity**: Studies with **SPARX** were tailored to the profiles/needs of sexual minority youth (Lucassen, 2015) and cultural minority youth (Shepherd, 2018); also, youth with high mental health needs, those socially and economically disadvantaged or excluded from mainstream education and/or early school leavers (Fleming, 2011; Kuosmanen, 2017); equity testing provided for internet access (Jander, 2016).  **Engagement**: (See Table 3)  **Ethical practices**: eight studies noted important ethical enhancements: four studies set out specific safety measures and protections against high depression/anxiety or risk of self-harm (Kuosmanen, 2017; Lucassen, 2015; Merry, 2012; Stasiak, 2014). Fleming, 2011 noted that all students (study participants or not) played **SPARX** to spare participants the embarrassment of being seen as depressed or needing help. Two studies provided letters to participants, assuring them that their data would not be shared (Jander, 2016; Scholten, 2016). Therapists reporting on female adolescents in their practices cautioned that **Maya** may not be appropriate for patients already addicted to video games (Carrasco, 2016).  **YOUNG ADULTS**  **Processes**: This group of studies included two RCTs (Pinto, 2015; Poppelaars, 2021) and one clinical trial (Hwang, 2021), two pre-post game assessments (Kreutzer, 2015; Hong, 2018), one mixed methods (Olivet, 2019), and two single case studies (Botella, 2011; Fagundo, 2014). Barriers: study design issue: a study design/research issue involving burdensome travel requirements to the study site that affected study feasibility (Pinto, 2015).  **Impact**: All eight were empirical studies, five interventions showing improvement on the main variables of interest: on fear/avoidance in the **Cockroach Game (**Botella, 2011): on binge eating in **PlayMancer (**Fagundo, 2014); on video game self-efficacy and attitudes to game in **Walk in My Shoes**: Kreutzer, 2015; in attitudes toward recovery in **On Track>The Game**: Olivet, 2019 and on game feasibility (vs. controls) in **e-SMART-MH:** (Pinto, 2015). For the commercial game, **Journey** (Poppelaars, 2021), no positive effects on depressive symptoms; 50.2% still had depressive symptoms at follow-up.  Secondary measures (e.g., life satisfaction, quality of life, other psychological functioning variables; perceived stigma, economic outcomes) NA.  **Effectiveness**: described/demonstrated for the **Cockroach Game** (Botella, 2011), with the largest effect on avoidance, then fear, but no impact on related irrational thoughts; and for **OCfree** (Hwang, 2021), shown to be as effective for treating OCD as traditional offline CBT. **Journey**, a stand-alone commercial game (Poppelaars, 2021) was tested for therapeutic use in reducing depressive symptoms. Results showed no effectiveness for use as a depression prevention strategy.  User satisfaction/acceptability: **On Track>The Game**: game enhanced recovery, hope, confidence in treatment; users and providers appreciated game interactivity (customizing characters, planning day, visiting locations); video testimonies of mental health recovery were greatly appreciated; view that game would be helpful in first episode psychosis, and may assist personal decision-making (Olivet, 2019); **e-SMART-MH**: rated acceptable, virtual coach and content (players wanted more use of Artificial Intelligence and self-tailoring) (Pinto, 2015); **OCfree**: satisfaction 3.4/5; 50% would continue using program and 70% recommended it (Hwang, 2021); **Cockroach Game**: player found game very helpful. Adherence rates: 91.4% (Hwang, 2021), 46.7% (Pinto, 2016) and 93.9% (Poppelaars, 2021). Studies that did not report retention data were: Hong, 2018; Olivet, 2019; Fagundo, 2014; Kreutzer, 2015; and Botella, 2011. The single patient with specific phobia was able to use the intervention daily with decreasing anxiety (Botella, 2011).  **Sustainability**: Only the two single case studies measured sustainability at follow-up, both showing that gains were maintained or improved at 12-month follow-up: The **Cockroach Game (**Botella, 2011) and **PlayMancer (**Fagundo, 2014). Two other studies provided anecdotal information about willingness of participants to access the intervention after the trial (Hwang, 2021; Pinto, 2015). The other four studies provided no information on sustainability.  **Equity:** The two US studies purposefully recruited a high percentage of ethnic minorities (African American, Hispanic, mixed race) (Olivet, 2019; Pinto, 2015).  **Engagement**: (see Table 3)  **Ethical practices**: Virtual reality exposure to phobia (here cockroaches) was considered more ethical than in vivo exposure, which purposefully evokes distress (Botella, 2011); there was no other commentary on ethics in this group of studies. |
| **Step 4: Severe and complex mental health conditions (7 games; 9/49 studies: 18.4%)** | | |
| **YOUTH** in inpatient psychiatry units for depression, suicidal ideation, and/or anger (Bobier, 2013; Ducharme, 2012)  **YOUTH** in inpatient units/outpatient clinics for substance use disorder, opioid addiction and/or marijuana use disorder (Abroms, 2015, 2019; Sanchez, 2015)  **YOUTH** in residential institutions for complex trauma and trauma-related conditions (PTSD, ADHD, attachment disorder; some comorbid Intellectual  Disability) (Aventin, 2014; Schuurmans, 2015, 2018, 2021)  **YOUNG ADULTS**:  None. | Therapeutic games:  **The SIMS Life Stories^TM^** (Aventin, 2014): Attachment, Self-regulation, and Competence model  **SPARX** (Bobier, 2013): Cognitive Behavioral Therapy (CBT)  **Muse** (Schuurmans, 2021): CBT  **RAGE-Control** (Ducharme, 2012): Anger Control Training, CBT  Relapse prevention games:  **Recovery Warrior** (Abroms, 2015): Social Cognitive Theory, Reinforcement Theory of Motivation; (Abroms, 2019): same as Abroms, 2015 + Repetition Priming, Social Learning  **Arise** (Sanchez, 2015): types-NA.  Games with biofeedback (adjunct to therapy):  **Dojo** (Schuurmans, 2015, 2018): Emotion Regulation Training; CBT techniques. | **Processes**: 3 RCTs (Abroms, 2019; Schuurmans, 2018, 2021), 1 open trial (Bobier, 2013), 2 pre-post game assessments (Abroms, 2015; Schuurmans, 2015), 1 pilot survey (Sanchez, 2015), 1 qualitative study (Aventin, 2014), 1 single case study (Ducharme, 2012) were described. Barriers: study design issues: re inpatient settings, lack of time, space, volatile schedule, other needs (Aventin, 2014); illness acuity, ward activities, intervention offered close to discharge (Bobier, 2013); challenges in recruitment of drug patients, higher doses of game needed (Sanchez, 2015; Abroms, 2019); active control group not allowed, as no mental health benefits expected. Both groups got treatment as usual, giving the **Muse** group extra attention (Schuurmans, 2021); staff issues: dedication to practical work, but little support for therapeutic work (Aventin, 2014).  **Impact**: Significant positive impact of game intervention on key variables in 3 of the 7 games: abstinence from drugs in **Recovery Warrior** (Abroms, 2019); anxiety and externalizing behavior in **Dojo** (Schuurmans, 2015, 2018), and in posttraumatic symptoms and stress, anxiety, aggression, and depression in **Muse** (Schuurmans, 2021).  Secondary measures (e.g., life satisfaction, quality of life, perceived stigma, economic outcomes; acceptability ratings): NA.  **Effectiveness**: described/demonstrated for **Dojo** and **Muse** in residential settings (Schuurmans, 2015, 2018, 2021).  User satisfaction: positive views of game for Abroms, 2019; Aventin, 2014. Increasing satisfaction over first 3 weeks; less resistance to treatment if game available; all players wanted to play weekly as part of treatment; some wanted to play daily (Abroms, 2015); social workers saw game as a tool for engaging residents in the therapeutic world and for building therapeutic relationships (Aventin, 2014); 18/20 inpatients viewed **SPARX** as useful or very useful (Bobier, 2013); the single participant playing **RAGE-Control** rated four sessions as extremely helpful, one session neutral. Player felt the game enhanced her engagement in therapy (Ducharme, 2012); both adolescents and providers found **Arise** informative and engaging; they strongly recommended continuing game development; Re **Dojo**, high player satisfaction and enjoyment, for self and others; they liked that it was a video game intervention; **Dojo** seen as useful for daily life (Schuurmans, 2015, 2018); **Muse** enjoyed more by male vs. female players and intervention effect was stronger for males (Schuurmans, 2021).  Acceptability: 93% thought **SPARX** would appeal to others; 79% would recommend it to friends (Bobier, 2013); strong acceptability ratings (users and providers) for **Arise** (Sanchez, 2015); self-efficacy: self- efficacy scores on resistance to marijuana constant after four weeks, and 44.4% abstinent with **Recovery Warrior** (Abroms, 2015); less externalizing behavior and less anxiety based on user and caregiver reports noted for **Dojo** (Schuurmans, 2015; 2018). Program completion rates (adherence) were: 66.7% (Abroms, 2015); 36.7% (Aventin, 2014); 80.0% (Abroms, 2019); 10.0% (Bobier, 2013); 90.0% (Schuurmans, 2018), 77.7% (Schuurmans, 2021) and 100.0% (Sanchez, 2015); NA for Schuurmans, 2015; Ducharme, 2012.  **Sustainability**: Of five studies with positive impact, gains carried over to follow-up in three studies, **Arise** (Sanchez, 2015), **The** **SIMS Life Stories^TM^** (Aventin, 2014) and one study with **Dojo** (Schuurmans, 2015).  **Equity**: lack of testing or mental health treatment in youth residential homes was critiqued (Aventin, 2014); one game highlighted the need for personalization regarding racial diversity, socioeconomic status, and reading levels (Sanchez, 2015).  **Engagement**: (See Table 3)  **Ethical practices**: Consent procedures applied in all studies; further enhancements reported in three studies: restricting data-sharing with parents/clinicians to usage data only (Sanchez, 2015); restoration of trauma therapy before FU due to vulnerability of participants, and private data storage (Schuurmans, 2021); suggestion that video games may substitute for use of seclusion and restraints to control aggressive behavior (Ducharme, 2012). |
